# Risk-Aware Model-Based Control

**DOI:** 10.3389/frobt.2021.617839

**Published:** 2021-03-11

**Authors:** Chen Yu, Andre Rosendo

**Affiliations:** Living Machines Laboratory, School of Information Science and Technology, ShanghaiTech University, Shanghai, China

**Keywords:** machine learning, reinforcement learning, dynamics model, risk awareness, conditional value at risk, data efficiency, eidos, mujoco

## Abstract

Model-Based Reinforcement Learning (MBRL) algorithms have been shown to have an advantage on data-efficiency, but often overshadowed by state-of-the-art model-free methods in performance, especially when facing high-dimensional and complex problems. In this work, a novel MBRL method is proposed, called Risk-Aware Model-Based Control (RAMCO). It combines uncertainty-aware deep dynamics models and the risk assessment technique Conditional Value at Risk (CVaR). This mechanism is appropriate for real-world application since it takes epistemic risk into consideration. In addition, we use a model-free solver to produce warm-up training data, and this setting improves the performance in low-dimensional environments and covers the shortage of MBRL’s nature in the high-dimensional scenarios. In comparison with other state-of-the-art reinforcement learning algorithms, we show that it produces superior results on a walking robot model. We also evaluate the method with an Eidos environment, which is a novel experimental method with multi-dimensional randomly initialized deep neural networks to measure the performance of any reinforcement learning algorithm, and the advantages of RAMCO are highlighted.

## 1 Introduction

The controllers of robots are primarily designed and tuned by human engineers through tiresome iterations and require extensive experience and a high degree of expertize (Deisenroth et al., 2013). The resulting programmed controllers are built upon assuming rigorous models of both the robot’s behavior and its environment. As a consequence, hard-coded controllers for robots have its limitations when a robot needs to adapt to a new situation or when the robot/environment cannot be precisely modeled. Machine learning and, particularly, deep learning, have made ground-breaking success in various domains, such as speech recognition (Hinton et al., 2012), computer vision (Krizhevsky et al., 2012), video games (Mnih et al., 2015), or medicine (Xu et al., 2020) in recent years. However, unlike other machine learning branches, RL is still not widely applied to real-world engineering products, especially in the field of robotics. Overall, the main obstacles on the application of RL to such problems are 1) data inefficiency, 2) lack of robustness, and 3) lack of practical advantage over hand-tuned controllers.

It can be easily stated that most of the successful and famous methods up to now (Lillicrap et al., 2015; Schulman et al., 2015, 2017; Haarnoja et al., 2018) require millions of steps to find the best policy, which is acceptable in simulators but impractical in a real-world application. Data-efficiency is an important consideration when applying machine learning techniques in a real robot (Chatzilygeroudis et al., 2020). Unlike applications in video games or images processing, it is unrealistic to train a robot in the real world with millions of trials, since implementation in real-world can suffer from mechanical wearing and prohibitive wall-clock time.

Safety is another consideration when it comes to some applications, such as self-driving cars, surgical robots, or assistive robots. In these applications, the loss is not only a number like in a simulator but a clear threat to human life. The finance literature differentiates between three risk-related types of behavior, namely risk-neutral, risk-averse and risk-seeking. Decision making by RL agents typically involves the optimization of a risk-neutral performance objective, namely the expected total discounted reward. This approach neither takes into account the variability of the cost or the modeling errors and hence becomes another barrier for the application of machine learning on robotics. Furthermore, the gap between implementation and mathematical theories shown by Engstrom et al. (Engstrom et al., 2020) reveals the unreliability of using RL in risk-sensitive problems, which implies that decision-making algorithms should be more conservative when they are designed for real-world applications.

The current state-of-the-art for robotic control is vastly model-based and human-designed, with simulators being used to calculate paths and find solutions before deployment (Mouret and Chatzilygeroudis, 2017). Unlike some applications such as playing video games, it is very difficult for an RL agent to produce better performance than human experts. While most of the successful learning-based controllers still produce peculiar locomotion gaits on legged robots, the most impressive works in robot control do not include machine learning algorithms for control design (Fabisch et al., 2019; Kim et al., 2020). We claim that the learning-based controllers can be essential and outperform human-designed controllers when the agent faces an unknown environment, where even humans would not know the optimum, instead of learning trivial control tasks.

The motivation behind this work is to take a step towards narrowing the gap between academic advances and industrial applications by handling the aforementioned problems. First, we focus on model-based reinforcement learning (MBRL) with a probabilistic dynamics model, as incorporating uncertainties into our dynamics model usually prevents overfitting with insufficient data. Second, we consider the risk based on this uncertainty to protect real-world applications from dramatic loss due to model uncertainty. Prior works on Robust Markov Decision Processes have traditionally dealt with risk due to uncertainty in the transition and reward parameters (Givan et al., 2000; Mannor et al., 2012). However, most of these works assume inherent stochasticity of the environment, while model uncertainty due to lack of data should, in fact, be regarded as a bigger issue from an engineering standpoint. Finally, we expect that the RL environment in our simulator can equally evaluate the performance of the agent in real-world applications, even dealing with some unknown problems.

Our primary contribution is a novel MBRL algorithm called Risk-Aware Model-Based Control (RAMCO). We employ model-free solvers to produce warm-up data, train a Bayesian dynamics model based on these data, and do the planning based on Conditional Value at Risk (CVaR) measurement. The overall results are shown to be competitive when compared to other successful state-of-the-art RL algorithms. In addition, to better evaluate and compare the performances of different RL algorithms, we propose a real-world-inspired walking robot model called AntX and a novel pseudo-environment method called Eidos that can emulate an RL environment with any complexity.

## 2 Related Work

**FIGURE 1:**
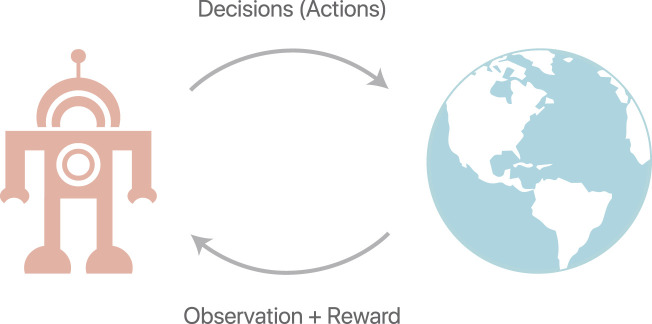
An illustration of the interaction within an RL agent and its environment.

Reinforcement Learning has contributed to the machine learning community with a wide variety of applications, ranging from robotics to finance. It is a computational approach for solving goal-oriented decision-making problems (Sutton and Barto, 2018), described as a process of interaction between an agent and an environment (Figure 1).

Most current research on RL is built on the theoretical framework of Markov Decision Process (MDPs) (Puterman, 1994), which is a general formalism for the study of decision-making problem. In this classic theoretical framework, an agent takes actions typically in a discrete-time sequence. In each time step, it is said that the agent is in a state, representing the information needed from the environment. Then according to a policy, an agent would take a specific action in each state. After that, the agent would be transited into the next state and receive a reward signal. The unknown function that map a tuple (state, action) to the next state is usually called the transition function. Some algorithms try to model this transition function as a dynamics model and hence are called model-based method.

### 2.1 Model-free Reinforcement Learning

Popular RL algorithms, such as Deep Q learning and Deep Deterministic Policy Gradient (DDPG) (Lillicrap et al., 2015), do not assume any transition model on the environment, and hence they can also be called model-free methods. These methods have shown great promise as a general-purpose solver for complex learning problems. We take some other popular advanced model-free algorithms as examples: Distributed Distributional Deterministic Policy Gradients (D4PG) (Barth-Maron et al., 2018) is a variant of DDPG, and it uses multiple actors to collect samples in parallel and store the collected data into a shared replay buffer. A distributional value function is used in the Critic part, while experiment shows that this trick improves the performance. Twin Delayed Deep Deterministic policy gradient (TD3) (Fujimoto et al., 2018) is another variant of DDPG, using two critic networks to overcome the overestimation bias. Instead of using Q function Q(s,a) in the Critic part as DDPG, D4PG or TD3, the method of Advantage Actor-Critic (A2C) (Wu et al., 2017) uses a Value function V(s) in the Critic. A distributed version of A2C called Asynchronous Advantage Actor-Critic (A3C) improves both the performance and training speed. Trust Region Policy Optimization (TRPO) (Schulman et al., 2015) also uses a Value function V(s) in the Critic. However, although TRPO could be considered as having an Actor-Critic structure, the Actor is not updated according to the gradient from the Critic as other Actor-Critic methods mentioned before but updated like other policy-based methods. It utilizes the Critic component for calculating an Advantage function and uses KL-divergence metrics to control the update rate of the policy. Proximal Policy Optimization Algorithms (PPO) (Schulman et al., 2017) is a simplified version of TRPO, replacing the KL-divergence function as a clipping function to also control the update rate. Soft Actor-Critic (SAC) (Haarnoja et al., 2018) is another model-free Actor-Critic method, adding entropy of the policy as part of its objective function. This allows for a better exploration of the training process. Overall, model-free methods are popular because of their scalability, which makes it a promising tool for many high-dimensional tasks (Osiński et al., 2020; Schoettler et al., 2020; Ye et al., 2020). However, as we will discuss in Section 7.1, data inefficiency is a common problem for model-free methods.

### 2.2 Model-Based Reinforcement Learning

Compared with model-free approaches, MBRL algorithms are generally considered as being more data-efficient since they are able to model the transition function and hence train on the modeled environment. Once the transition function is learned, the reinforcement learning problem indeed becomes a Dynamic Programming function. MBRL methods could be categorized based on whether an explicit policy exists. The method of Probabilistic Ensembles with Trajectory Sampling (PETS) (Chua et al., 2018) is a recent successful example for the ones without a policy. It uses a deep neural network with ensembles (Lakshminarayanan et al., 2017) to model the environment dynamics taking the uncertainty in consideration, and does open-loop planning on this model. The PETS method is also successfully applied to real robots in (Nagabandi et al., 2019). The model-based control method introduced in (Nagabandi et al., 2018) is similar, but with a deterministic dynamics model. Deep Planning Network (PlaNet) (Hafner et al., 2019) is another successful method without policy. It uses a recurrent state-space model and shows its advantage of image-based control. As for MBRL with an explicit policy, Probabilistic Inference for Learning Control (PILCO) (Deisenroth and Rasmussen, 2011) is one of the most popular methods. It uses Gaussian Process to model the transition function of the environment and lowers the model bias by taking the uncertainty of the input into consideration, leading to a more accurate long-term prediction. Cutler et al. successfully applied the PILCO method to an inverted pendulum model (Cutler and How, 2015) and Englert et al. applied it to an imitation learning problem (Englert et al., 2013). Nonetheless, PILCO relies on Gaussian Process, which limits its applicability for complex problems that need more trials to be solved. Furthermore, ignorance of the temporal correlation in model uncertainty between successive states can lead to underestimation of state uncertainty at future time steps (Deisenroth et al., 2015). Deep PILCO (Gal et al., 2016) is proposed to make up for these drawbacks by replacing the Gaussian Process component with a Bayesian neural network dynamics model but still suffers from lack of scalability.

### 2.3 Combination of Model-free and Model-Based Methods

Many previous works also try to combine advantages from both model-free and model-based RL. A control pipeline proposed in the work (Nagabandi et al., 2018) uses random-shooting and model predictive control (MPC) method to find an optimal action at each state based on a dynamics model and then produces multiple sub-optimum rollouts. These rollouts are severed as training data for a neural network to learn a closed-loop policy, using a DAGGER (Ross et al., 2011) method. Finally, the weights of this network are used as the initialization of the policy network in a TRPO method. It is shown that this framework can improve the data efficiency of pure model-free methods. Another way for hybridizing model-based and model-free methods is Dyna algorithm, which uses a model to generate synthetic samples for model-free policy optimiser. The original Dyna-Q algorithm (Sutton, 1990) use a model to have a better Q function estimation based on Q-learning method. Recently proposed Dyna-style methods including Model-Based Acceleration (MBA) (Gu et al., 2016), Model-Based Value Expansion (MVE) (Feinberg et al., 2018), Model-Based Policy Optimization (MBPO) (Janner et al., 2019) and so on. These methods usually differ from whether the training is based on real-world rollout or predicted rollout, or whether using predicted rollouts to estimate target value or to train the whole Q function. The experiment of the MBPO method shows that it can obtain better sample efficiency than prior model-based methods and asymptotic performance of the state-of-the-art model-free algorithms.

### 2.4 Risk-Sensitive Reinforcement Learning

There is another aspect we can evaluate an RL method: whether or not it is risk-aware. All the works we have mentioned above are risk-neutral. There are also works applying reinforcement learning to robust MDP setting, which has traditionally dealt with risk due to uncertainty in the transition and reward parameters. Tamar et al. (Tamar et al., 2014) use approximate dynamic programming (ADP) to scale up the robust MDP paradigm with larger state space. Roy et al. (Roy et al., 2017) propose a robust counterpart to Q-Learning, which is robust to model misspecification, while Derman et al. (Derman et al., 2019) propose a robust version of DQN for higher dimensional domains. Tessler et al. (Tessler et al., 2019) propose a robust variant of DDPG, training two deterministic policy networks, the Actor and the Adversary. The Adversary is a potentially adversarial policy. Similar to DDPG, a critic is trained to update the joint-policy. It is shown that robust-oriented RL can do better in generalization, although it is still not clear how the connection between robustness and generalization holds in RL (Tessler et al., 2019). While the works mentioned above are dealing with uncertainty due to inherent stochasticity of the environment, a. k.a. aleatory uncertainty, it is usually not the most important source of risk occurs in engineering. It is because systems in engineering are nearly deterministic, and most uncertainty is due to the lack of information about the environment (Eriksson and Dimitrakakis, 2020). Therefore, Depeweg et al. (Depeweg et al., 2017) focus on decomposition in aleatory and epistemic risk. They employ a Bayesian neural network to model the underlying dynamics and utility function to do a trade-off between expected return and each risk. The work by Eriksson et al. (Eriksson and Dimitrakakis, 2020) is also developed in a Bayesian framework, using policy gradient and utility function to leverage preferences between risk and expected return based on a model-free setting.

## 3 Preliminaries

We formularize our control problem as a continuous state space Markov Decision Process, written as a tuple $M=\left(\text{S,A},r,\mathbb{P},\gamma ,d_{0}\right)$, where $\text{S}\in {\mathbb{R}}^{d_{s}}$ and $\text{A}\in {\mathbb{R}}^{d_{a}}$ are the sets of states and actions; $r_{t}\left(s_{t},a_{t}\right)$ is a deterministic cost, which describes the task and hence previously known. $\mathbb{P}\left(\cdot |s_{t},a_{t}\right)$ is the transition probability distribution, from which each next state is sampled: $s_{t+1}∼\mathbb{P}\left(\cdot |s_{t},a_{t}\right)$. γ∈[0,1) is a discounting factor and $d_{0}$ is the distribution of the initial state $s_{\text{0}}$. The goal of our RL algorithm is to learn a policy that maximizes the total rewards within a given time period. At each time step *t*, the agent is in state $s_{t}\in \text{S}$, takes action $a_{t}\in \text{A}$, receives reward $r_{t}\left(s_{t},a_{t}\right)$ , and is transited to next state $s_{t+1}$, following an unknown transition probability distribution $\mathbb{P}\left(\cdot |s_{t},a_{t}\right)$. The objective at each time step is to execute any action that maximizes the discounted sum of future rewards $V_{t}$.

In MBRL, the transition function is usually expressed as a dynamics model. Throughout this paper, we consider a dynamics model $\hat{f}\left(\cdot \right)$ as in the equation:

$${\hat{s}}_{t+1}=s_{t}+\hat{f}\left(s_{t},a_{t}\right).$$ (1)

**FIGURE 2:**
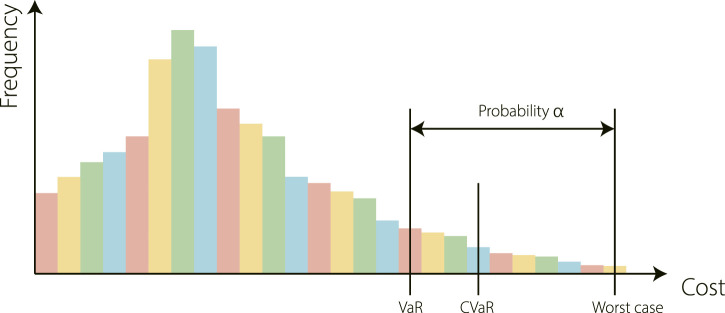
An example to illustrate three risk metrics: Value at Risk (VaR), Conditional Value at Risk (CVaR), and the worst case. Intuitively, Var is the (1−α) quantile of the cost distribution, and CVaR is the expected value of the cost distribution’s upper (1−α) tail.

The dynamics model does not directly output the predicted successive state ${\hat{s}}_{t+1}$ because this can be challenging when the states ${\text{s}}_{t}$ and the corresponding next states $s_{t+1}$ are too similar (Nagabandi et al., 2018). We further define the transition function as a Gaussian Process, and consider that this can capture both aleatoric uncertainty and epistemic uncertainty. It means that we can not only take into account uncertainty caused by a noisy environment but also uncertainty arises from the fact that data is not sufficient to determine the underlying parametrization of a model uniquely. Furthermore, we consider risk-averse reinforcement learning, whose risk is assumed to come from these uncertainties. We regard the negative of total discounted future reward as loss and use the risk-measure method Conditional Value at Risk (CVaR) (Righi and Ceretta, 2016). It is a variant of Value at Risk (VaR) (Mansour and Abdel-Rahman, 1984) and also recently identified as suitable for measuring risk in robotics (Majumdar and Pavone, 2020). For a specific random variable *Z* and a confidence level α, the CVaR is the expected loss in the worst α cases, illustrated as Figure 2. It is calculated according to Eq. 2 for a random normal variable $X∼N\left(\mu_{h},\sigma_{h}^{2}\right)$.

$${\text{CVaR}}_{h,\alpha }\left(X\right)=\alpha^{-1}\varphi \left(\Phi^{-1}\left(\alpha \right)\right)\sigma_{h^{-}}\mu_{h},$$ (2)

In Eq. 2, φ(z) denotes the standard Gaussian probability density function and $\Phi^{-1}\left(\alpha \right)$ is the α quantile of the standard Gaussian distribution. In each action $a_{t}$ at time step *t*, we do moment-matching over the distribution of the accumulated future reward and calculate the corresponding CVaR with this equation.

Overall, we want to find an (implicit) policy π that can maximize the cumulative reward starting from the first state $s_{0}$ towards a final state $s_{T}$:

$$J^{\pi }={\text{argmax}}_{\pi }\sum_{t=0}^{T}r\left(s_{t},a_{t}\right).$$ (3)

The transformation of this objective function to relate it with the risk evaluation functions would be discussed in the next section.

## 4 Risk-Aware Model-Based Control

**FIGURE 3:**
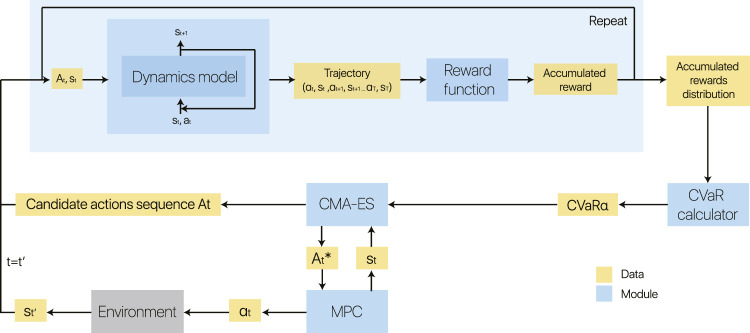
Block diagram of our model-based reinforcement learning method on run time. For each state $s_{t}$ at time step *t*, we obtain a list of candidate actions sequence $A_{t}$ from the CMA-ES optimiser. We choose the best actions sequence $A_{t}^{⋆}$ from this list by calculating the CVaR value of each choice. To obtain this CVaR value, we use a Bayesian neural network (BNN) to predict a sufficient number of trajectories for modeling distribution of accumulated rewards. After the best actions sequence $A_{t}^{⋆}$ is found, the first action in this actions sequence is truly performed by the agent (model predictive control, MDP). Training of the BNN dynamics model is ignored in this block diagram for simplicity. A more detailed description of the system can be seen in the pseudocode Algorithm 1.

In this work, we propose a model-based method with a probabilistic dynamics model, and our main objective is to learn a safe and scalable policy efficiently. We combine it with an MPC, taking a CVaR into consideration, to perform planning using this dynamics model. Overall, the core contribution of this work is a risk-sensitive model-based control framework, which uses a Bayesian neural network to capture aleatoric and epistemic uncertainty and uses CVaR to trade between risk and return. The overview of our method is illustrated in Figure 3. We now detail our control framework.

### 4.1 Dynamics Model: Transition Function as a Gaussian Process

*First, predicting the next state.* Any MBRL algorithm has to select some mechanism to model the environment dynamics. This selection is generally of significance for an MBRL algorithm, since even small error can considerably affect the quality of the corresponding controlled results (Abbeel et al., 2006). There are lots of choices for the function $\hat{f}\left(\cdot \right)$ in Eq. 1. However, common deterministic approximators such as deep neural network are not enough for our application. For our data-efficiency and robustness expectations, we regard the function as a Bayesian neural network, which is a probabilistic model that places the flexibility of deep neural network in a Bayesian framework (Blundell et al., 2015), where the weight of each hidden unit is represented by a distribution. In practice, there are various ways for the approximation of this structure, since the accurate form is too complex to be tractable in a computer (Blundell et al., 2015). In this work, we adopt the Monte Carlo dropout method (Gal and Ghahramani, 2016) for an approximation of this structure. Since our dynamics model $\hat{f}$ can be explained as a Gaussian process, the problem can be described as: given a training dataset consisting of *N* state-action pairs $\left\{{\left(s_{t},a_{t}\right)}_{1},\ldots ,{\left(s_{t},a_{t}\right)}_{N}\right\}$ and their corresponding outputs $\left\{{\left(\text{Δ}s_{t+1}\right)}_{1},\ldots ,{\left(\text{Δ}s_{t+1}\right)}_{N}\right\}$, we are trying to search for a function $y=\hat{f}\left(\cdot \right)$ which is prone to have generated these observations. According to (Gal and Ghahramani, 2015), this objective can be approximated as Eq. 4 with *K* times sampling, where $M_{1}$, $M_{2}$, and m are weights with dropout probability $p_{1}$ and $p_{2}$.

$$J_{VI}\propto -k\overset{K}{\underset{n=1}{\sum }}{\left|\left|\text{Δ}s_{t+1,\text{n}}-\text{Δ}{\hat{s}}_{t+1,\text{n}}\right|\right|}_{2}^{2}-\frac{p_{1}}{2}{\|M_{1}\|}_{2}^{2}-\frac{p_{2}}{2}{\|M_{2}\|}_{2}^{2}-\frac{1}{2}{\left\|m\right\|}_{2}^{2}.$$ (4)

This means that we can implement this stochastic dynamics model based on a structure of deep neural networks, with dropout layers. Compared to common deterministic neural networks, all the dropout layers are not only activated during the back-propagation phase but also when making an inference.

### 4.2 Training the Dynamics Model

#### 4.2.1 Training Data Collection and Pre-processing

Initially, we collect warm-up training data by having agents take actions from a sampled initial state $s_{0}$, following a given policy $\pi_{\text{warm}-\text{up}}$. This can be a random policy or a policy with an off-the-shelf model-free agent runs from scratch. Theoretically, Soft Actor-Critic (SAC) (Haarnoja et al., 2018) algorithm would be a good choice since it will maximize a trade-off between expected return and entropy, producing better-explored data for feeding our dynamics model. We also employ Proximal Policy Optimization (PPO) as the warm-up policy in the experiment, which is one of the most successful model-free Policy Gradient methods. And then the resulting trajectories by $\pi_{\text{warm}-\text{up}}$ or the data generated by the training process of it are recorded, and denoted as trajectories $\tau_{T}=\left(s_{0},s_{0},s_{1},s_{1},\cdots ,s_{T-1},s_{T}\right)$ with episode length *T*. These data are then used for training dynamics model. We then slice the collected trajectories $\tau_{T}$ into state-action pair $\left(s_{t},a_{t}\right)$ at each time step *t* and their corresponding output label $\left(s_{t+1}-s_{t}\right)$. We then normalize the data to ensure the loss function weights different dimensions of the state equally. Zero-mean Gaussian noisy signal can also be added to the training dataset to increase the robustness of the model. We denote the training dataset as D.

#### 4.2.2 Model Training

To optimize the objective function (4), we build a network with three dense layers and dropout layers. In the *l*-th pair of layers, we have $w_{l}$ many units of W sampled from $m_{l}$ many units of M, according to a unified Bernoulli distribution with probability $p_{l}$ (a.k.a. dropout probability). Finally, we add the L2 regularization terms of each layer in the loss function, weighted by some weight decay lambda λ, leading to a sum of squared errors (SSE) loss function:

$$\varepsilon =\sum_{n=1}^{N}\Delta {\left\|s_{t+1,n}-\Delta {\hat{s}}_{t+1,n}\right\|}_{2}^{2}-\lambda_{1}{\left|\left|M_{1}\right|\right|}_{2}^{2}-\lambda_{2}{\left|\left|M_{2}\right|\right|}_{2}^{2}-\lambda_{3}{\left|\left|m\right|\right|}_{2}^{2}.$$ (5)

Note that the optimization objective of our network, which is just the minimization objective of the loss function Eq. 5 and Eq. 4 would both converge to the same limit. We minimize the loss function using an Adam optimiser.

#### 4.2.3 Model Validation

For validation, similar to some other supervised learning method in machine learning, we can calculate the mean square error (MSE) between the expectation of the predicted result and the true data given by a validation data set:

$$\varepsilon_{val}=\frac{1}{N_{val}}\sum_{n=1}^{N_{val}}{\left\|s_{t+1,n}-E\left(s_{t+1,n}\right)\right\|}_{2}^{2},$$ (6)

where $N_{val}$ denotes for the size of validation dataset, which is made up of trajectories not existed in the training dataset. We use Monte Carlo sampling for the calculation of the expectation term. The problem of this error evaluation is that it only takes one-step error calculation, whilst our goal is to predict a multi-step trajectory toward the future. Hence instead of calculating step-wize error, we evaluate the sequence-wize error through propagating the learnt dynamic model forward $T_{val}$ many times. Given a random actions sequence $\left(a_{t},\cdots ,a_{t+T_{val}-1}\right)$ from validation data set, we compare the corresponding ground-truth states sequence $\left(s_{t+1},\cdots ,s_{t+T_{val}}\right)$ with multi-step predictions results $\left({\hat{s}}_{t+1},\cdots ,{\hat{s}}_{t+T_{val}}\right)$ from the dynamics model, formulated as:

$$\varepsilon_{val}=\frac{1}{N_{val}}\sum_{n=1}^{N_{val}}\frac{1}{T_{val}}\sum_{t^{\prime }=1}^{T_{val}}{\left|\left|s_{t+t^{\prime },n}-E\left({\hat{s}}_{t+t^{\prime },n}\right)\right|\right|}_{2}^{2},$$ (7)

where the expectation of state $E\left({\hat{s}}_{t+t^{\prime }}\right)$ is calculated by *K* times Monte Carlo sampling:

$$E\left({\hat{s}}_{t+t'}\right)=\frac{1}{K}\sum_{k=1}^{K}\left(E\left({\hat{s}}_{t+t^{\prime }-1}\right)+\hat{f}\left(E\left({\hat{s}}_{t+t^{\prime }-1}\right),a_{t+t^{\prime }-1}\right)\right).$$ (8)

### 4.3 Policy Evaluation

*Then, evaluating the predicted consequences.* While this is a stochastic dynamic model, in each state, we are able to generate *K* next state samples, moment-match these samples as a Gaussian distribution, and sample from this distribution. Previous works show that this moment-matching could benefit data efficiency by penalizing multi-modal distributions through smoothing of the loss surface (Deisenroth and Rasmussen, 2011). The step-wize prediction can hence be expressed as

$$E\left({\hat{s}}_{t}\right)=\frac{1}{K}\sum_{k=1}^{K}\left(E\left({\hat{s}}_{t-1}\right)+\hat{f}\left(E\left({\hat{s}}_{t-1}\right),a_{t-1}\right)\right).$$ (9)

Given a sequence of actions starting from step *t*: $A_{t}=\left(a_{t},\cdots ,a_{t+H-1}\right)$ with horizon *H*, the dynamics model could generate a trajectory by recursively repeat this prediction process. With a given reward function, the accumulated rewards over horizon *H* is calculated, denoted as $V_{t,m}^{A_{t}}$. At each time step *t*, we sample *M*-many such trajectories. In our implementation, this trajectories sampling procedure is done in parallel. We then do a moment-matching again on these *M*-many $V_{t,m}^{A_{t}}$, force them to become a Gaussian distribution, and calculate the corresponding CVaR on this distribution. According to Eq. 2, the objective function Eq. 3 could then be rewritten as:

$$J^{A_{t}}={\text{argmin}}_{A_{t}}\left(\alpha^{-1}\varphi \left(\Phi^{-1}\left(\alpha \right)\right)\right)\left(\sigma_{V_{M}^{A_{t}}}-\mu_{V_{M}^{A_{t}}}\right)$$ (10)

with standard deviation $\sigma_{{\text{V}}_{M}^{A_{t}}}$ and mean $\mu_{{\text{V}}_{M}^{A_{t}}}$. Then, we want to find an optimal action sequence $A_{t}$ at each time step *t* that can minimize the CVaR of the total rewards predicted by our dynamics model. The parameter α in Eq. 10 represents the risk sensitiveness or risk preference of the agent. If the α is set to 0, it is equivalent to comparing the worst-case results among candidate actions, whereas the agent would be risk-neutral with an α of 1. In this work, we set the α as 0.05 as a trade-off between risk and return.

### 4.4 Optimization

*Finding the best action.* The calculation of the exact optimum of Eq. 10 can be challenging, as the transition and reward functions are both nonlinear. In this work, we use an evolutionary algorithm called Covariance Matrix Adaptation Evolution Strategy (CMA-ES) (Hansen, 2006). In each generation, it samples *N*-many candidate solutions from a multivariate Gaussian distribution N with mean vector m, covariance matrix C, and update step-size σ. For generation number g=0,1,2,⋯, the sampling process can be written as:

$$A_{t}∼N^{\left(g\right)}\left(m^{\left(g\right)},{\left(\sigma^{\left(g\right)}\right)}^{2}C^{\left(g\right)}\right).$$ (11)

Each candidate solution is evaluated according to the objective function (10). Parameters mean vector m and covariance matrix C are then updated to find a distribution $N^{\left(g+1\right)}$ that generates samples yield lower CVaR. In addition, the initialization method of the mean vector m depends on the choice of warm-up policy. If the policy $\pi_{\text{warm}-\text{up}}$ in the data-collection phase is a random policy, all m, σ, and C are initialized arbitrarily. While the policy $\pi_{\text{warm}-\text{up}}$ is by a model-free agent, we use this sub-optimum policy to produce a reference action sequence and use it to initialize the mean vector m. It means that each value $m_{i}$ in the mean vector m, which corresponds to each action $a_{t’}$ in the candidate action sequence $A_{t}$, is calculated by

$$m_{i}=a_{t^{\prime }}=\pi_{\text{warm}-\text{up}}\left(E\left(s_{t^{\prime }}\right)\right),$$ (12)

where the component $E\left(s_{t^{\prime }}\right)$ is calculated according to Eq. 9 recursively from $s_{t^{\prime }}=s_{t}$.

It was shown previously that CMA-ES could be served as an efficient tool for solving ill-conditioned functions and optimal control problems (Maki et al., 2020). For instance, to solve a quadratic function, it is proven that it can approximate the inverse Hessian empirically (Hansen and Auger, 2014) and theoretically (Akimoto, 2012). However, the problem of this method is that it would be computationally expensive for solving high-dimensional problems (10). Therefore, a variant of CMA-ES is used in our work. Inspired by VkD-CMA (Akimoto and Hansen, 2016b) which involves a simplification of the covariance matrix and an online-adapting simplification rule (Akimoto and Hansen, 2016a), we simplify the evolutionary path for computational efficiency.

Overall, we use a variant of CMA-ES as a black-box optimiser for the objective (10). For each generation, we sample *N* candidate solutions from a Gaussian distribution, calculate the CVaR of each candidate solutions, update the distribution and repeat.

### 4.5 Model-Based Control

We have shown how to solve Eqn. 10 and find an approximately optimal solution $A_{t}^{⋆}$ at time step *t*. Then, rather than having our agent take these sequential actions in open loop, we employ the MPC method: the agent executes only the first action $a_{t}$ for each optimized action sequence $A_{t}^{⋆}$. After receiving the next state signal $s_{t+1}$, it begins to re-plans for the next $A_{t}^{⋆}$ until an episode ends. Finally, the rollouts produced by the MPC controllers would be merged into the training data set   for retraining the dynamics model. In other words, the predictive model is also updated after the warm-up phase. This feedback of the dynamics model training process can narrow the gap between the states’ distribution of the training data and the true dynamics, and hence improve the performance of the dynamics model. When the new data set   obtained, based on which the warm-up policy $\pi_{\text{warm}-\text{up}}$ can then be updated. The pseudocode of our whole system in run time is shown as Algorithm 1. In the pseudocode, the best action over each iteration *t* in episode length *T* is taken for each time step. After *C* episodes, (i.e. rollouts) are completed, we update the training data set   and warm-up policy $\pi_{\text{warm}-\text{up}}$. This hyperparameter *C* can tune the update rate of the system to meet the users’ requirements of robustness.

## 5 Experimental Setup

### 5.1 AntX: A Real-World-Inspired Robot Model

The main robot model we use to test our RAMCO algorithm is AntX model, which is modified from the MuJoCo Ant-v2 benchmark model in OpenAI gym library. The original model contains a state space of 111 dimensions and action space of eight dimensions, while our model contains 29 dimensions of state space and also eight dimensions of action space. The modification is based on two reasons: First, we assume that the reward function r(s,a) is provided by the user before training and should be able to calculate from state s and a directly, which is a reasonable assumption in practice. Second is that some state dimensions defined in the original model are unavailable for a real-world robot. Finally, we tune down the control frequency to a ΔT of 0.2s and shorten each episode length *T* to 100 steps, both for practical consideration. It shares a similar reward function with Ant-v2 (ignoring the outer-force cost) and the same action space. Its state space is shown in Table 1.

**TABLE 1:** State space of our model AntX.

Dimension	Representation
0, 1, 2	Position (x, y, z) of the torso
3, 4, 5, 6	Orientation (x, y, z, w) of the torso
7, 8, 9, 10, 11, 12, 13, 14	Joint angels of all 8 joints
15, 16, 17	Directional velocity of the torso
18, 19, 20	Angular velocity of the torso
21, 22, 23, 24, 25, 26, 27, 28	Angular velocity of joints

**Algorithm 1:** Our algorithm RAMCO.

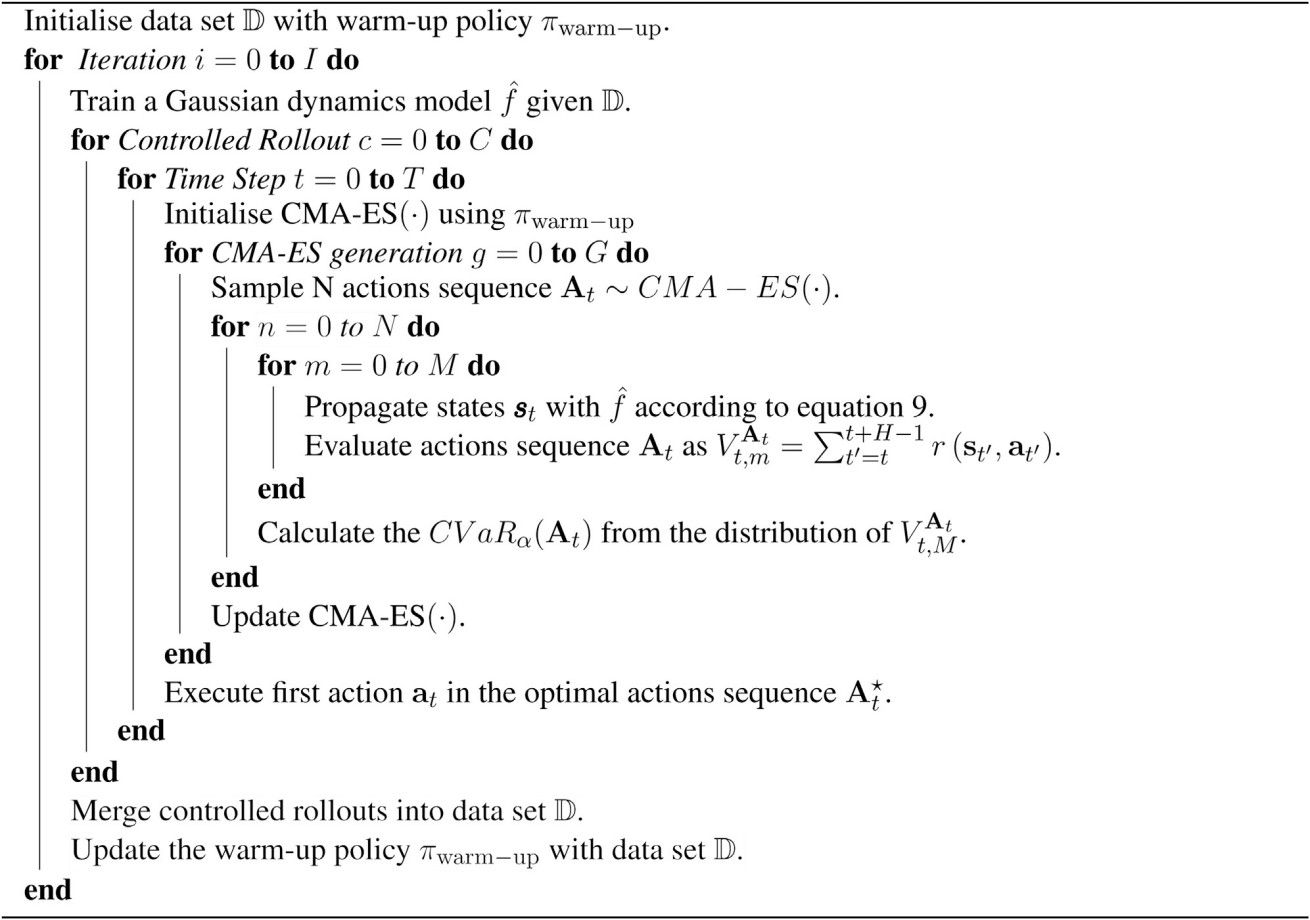

### 5.2 Eidos: A Pseudo Experimental RL Environment

As we claim that RL should show its advantage over solving unknown problems, we propose a novel experimental platform using a pseudo environment, named Eidos (a term coined by Plato as a permanent reality that defines a thing as what it is). We use Eidos to simulate an MDP with arbitrary state and action dimensionality. In this pseudo environment, we use a deep neural network with randomly initialized weights to represent the ground-truth dynamics function $f_{Eidos}\left(s\right)$, and another neural network with randomly initialized weights to represent the reward function $r_{\text{Eidos}}\left(s,a\right)$. Besides, this environment could also represent a POMDP problem, where observations are a sub-set of states.

**FIGURE 4:**
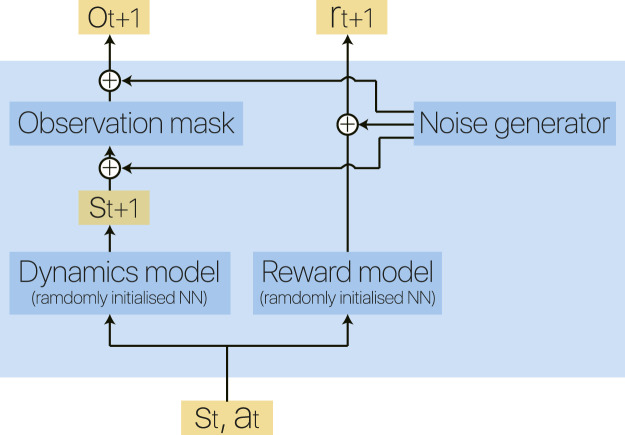
Block diagram of our Eidos environment. Two randomly initialized deep neural networks represent the dynamics model and reward model of an environment, making it flexible to vary the complexity of the corresponding MDP.

When the number of observation dimensions $d_{o}$ is chosen different from the of state dimensions $d_{s}$, a sub-space of $d_{o}$ dimensions would be sampled randomly without repeat from the state space. Furthermore, to emulate a noisy MDP, we consider reward, state and observation as signals and an additive white Gaussian noise (AWGN) is added to each with a Signal to Noise ratio (SNR) $SNR_{r}$, $SNR_{s}$, and $SNR_{o}$ respectively. The Eidos environment can be visualized in Figure 4. To the best of our knowledge, this is the first time that such an environment is proposed. We perform a case study on PPO to show its effectiveness on the evaluation of RL algorithms and show the results in Section 6. In the following experiments, we fix the action dimension to 10, and only the state is noisy with 60 SNR.

## 6 Experimental Results

In this section, we aim to answer the following questions:

- Can the dynamics model predict trajectories precisely?
- Is the Eidos environment effective as an evaluation method?
- How is the overall performance of RAMCO in terms of accumulated reward?

Our initial experiments aimed to test the trajectories from the dynamics model. Then, we stack up RAMCO against other state-of-the-art algorithms on a simulation of a walking robot. Finally, we briefly introduce our novel RL testing environment called Eidos and perform another comparison.

### 6.1 Dynamics Model

We first train the dynamics model on 11 benchmark environments and a simulated walking robot AntX (improved from Ant-v2) with the simulators MuJoCo (Todorov et al., 2012), PyBullet (Coumans and Bai, 2020), and Box2D (Catto, 2010), which are introduced in the supplementary material.

**FIGURE 5:**
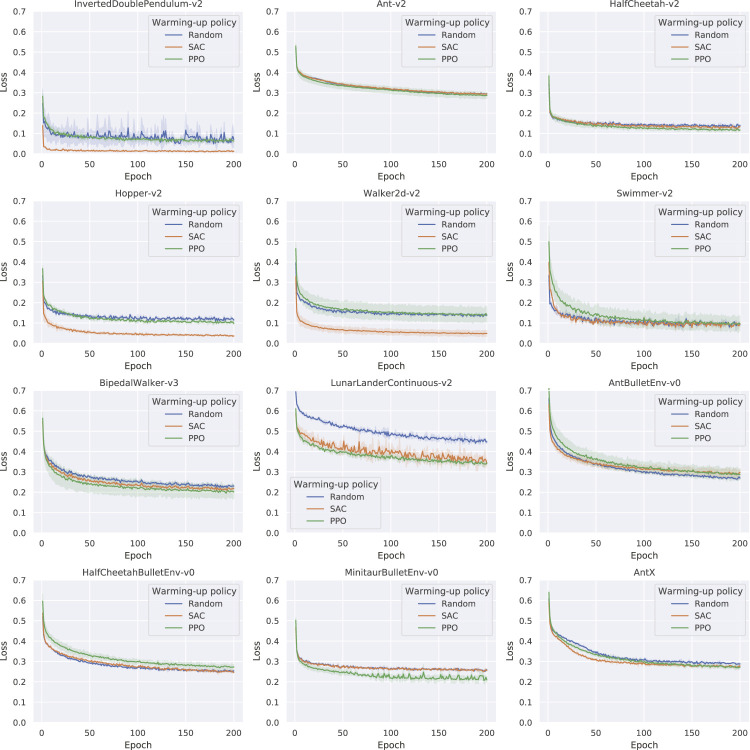
Learning curves of the dynamics model based on different warm-up data (Average of three trials), where the metric of loss is the sum of squared errors (SSE) with L2 regularization. In most cases, model-free methods significantly improve the dynamics model learning.

To estimate the effectiveness of using different policies as the back-end to generate training data, we compare training curves based on $10^{5}$ steps of warm-up data produced by different warm-up policies $\pi_{\text{warm}-\text{up}}$. Training loss described in Eq. 5 during the training process of the dynamics model is recorded. There are three different types of warm-up data sets for training the dynamics model: one generated by a random policy, one generated by SAC policy, and one generated by PPO policy. The two model-free policies are trained from scratch during the warm-up phase to generate the training data set, whose creation cost is also part of the cost of the warm-up for the whole RAMCO pipeline. As shown in Figure 5, we plot the training curves of the dynamics model based on different warm-up data. All the results data are the average results of three trials. We can obtain that for environments InvertedDoublePendulum-v2, HalfCheetah-v2, Hopper-v2, Walker2d-v2, BipedalWalker-v3, LunarLanderContinuous-v2, MinitaurBulletEnv-v0, and AntX, a model-free policy can produce warm-up data that reach a better training for the dynamics model compared to using random samples. We can also see that between two model-free policies, SAC showed a remarkable behavior in many warm-up trials, which agrees with the nature of SAC, a method that tries to maximize the entropy of the policy.

**FIGURE 6:**
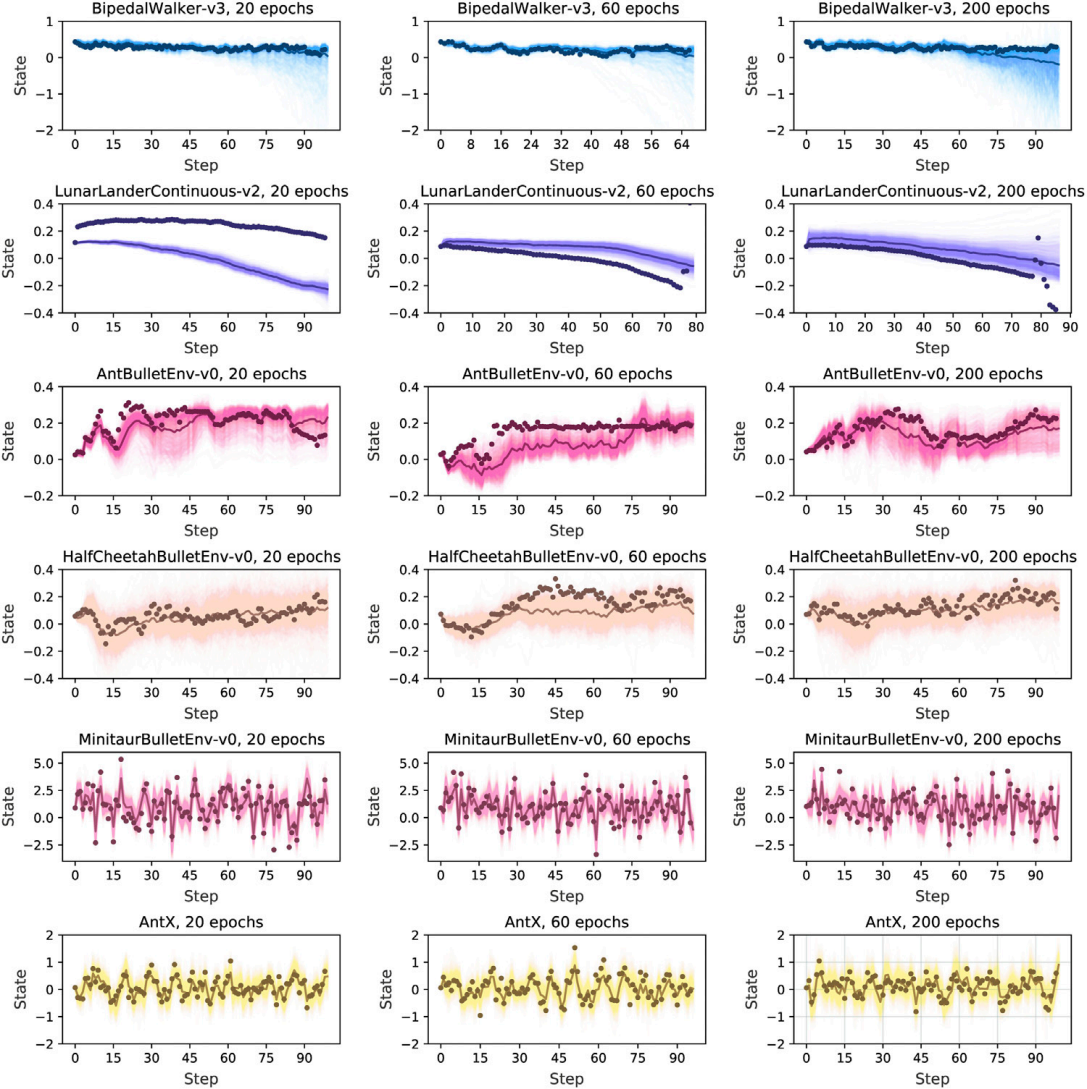
Predicted trajectories with RAMCO, with all dimensions of a state averaged over to a scalar. After a random warm-up we predict states recursively with the dynamics model. Each prediction and predictions average are in light and dark colors, and the baseline in dots. Through this visualization of predictive trajectories, we claim that this dynamics model meets the requirements of our control framework and can support our system to produce risk-aware control.

Based on the trained dynamics model, we then estimate the prediction performance of the model. We plot the output from the trained model with random warm-up data and with different training iterations (20, 60, 200). For visualization purposes, we average over all the dimensions of a state to a scalar. We generate a random sequence of actions and predict each state recursively with the dynamics model for 1,000 times. Some visualization results are shown in Figure 6 and the others are shown in the supplementary material. We can see a strong prediction from our algorithm with the baseline dots approaching the average prediction even when stretching the prediction horizon to 90 steps.

It is usually not good for a dynamics model if only the average output is close to the baseline but with high variance. This variance of prediction is unavoidable in real-world applications because of aleatory and epistemic uncertainty. For example, it is easy to observe from the case of BipedalWalker-v3 in Figure 6 that high prediction variance occurs when the prediction horizon is longer than 60 steps. However, our control framework can still deal with this variance in a risk-aware way. It is because we can observe from the figure that our sample-based dynamics model can always produce at least one accurate prediction. If this accurately predicted trajectory is dangerous, (i.e. with a very low return), our CVaR-based optimiser will take this into consideration and avoid performing the corresponding dangerous actions in the real world. In this case, our system can successfully detect and avoid the worst cases using CVaR, statistically eliminating risks and reducing losses, even though the predictions are noisy. Therefore, we claim that this dynamics model meets the requirements of our control framework and can support our system to produce risk-aware control.

**FIGURE 7:**
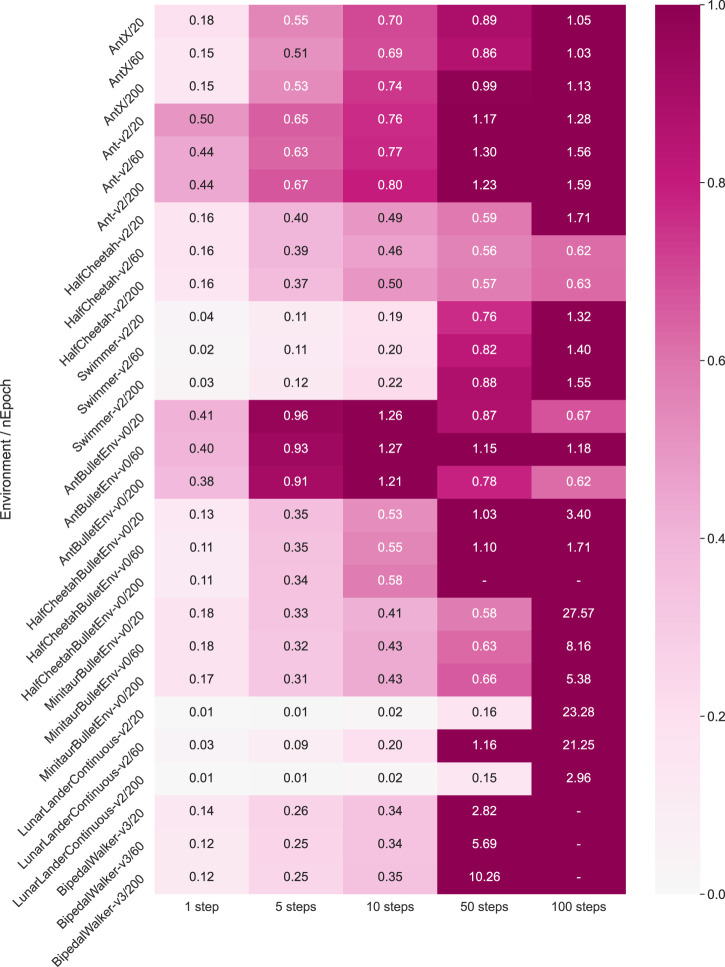
Dynamics model multi-step prediction error, with different RL environments (introduced in the supplementary material) and training epochs (20, 60, 200). We calculate the validation loss in Eq. 7 for one step, five steps, 10 steps, 50 steps, and 100 steps of prediction length. The smaller the value is, the more accurate the prediction makes, and the lighter color is shown. All the results are averaged after three trials.

In order to estimate the prediction accuracy quantitively and hence verify the effectiveness of our prediction, we then calculate the exact validation loss according to Eq. 7 for 1-step, 5-steps, 10-steps, 50-steps, and 100-steps prediction length $T_{val}$. This estimation is based on a dynamics model trained by a random warm-up data set, whose purposes are to 1) estimate the performance of the dynamics model numerically and 2) provide references for choosing an appropriate MPC horizon *H*. All the results are averaged from three trials and shown in Figure 7. As expected, long-horizon predictions concatenate more errors and training through more epochs reduce prediction errors. For some simple environments, such as Half Cheetah, the dynamics can even predict the state 100 steps ahead with an error lower than 1, while for complex environments, such as the Bipedal Walker, the error begins to surge beyond 50 steps. These results of static analysis of the prediction accuracy encouraged us to adopt a 10-step prediction as our control horizon *H*. With appropriate training parameters, this prediction accuracy will increase for each iteration *C* denoted in Algorithm 1 on the run, since the data distribution of training data set D will continuously become closer to the real-world trajectories distribution.

### 6.2 RAMCO on AntX

**FIGURE 8:**
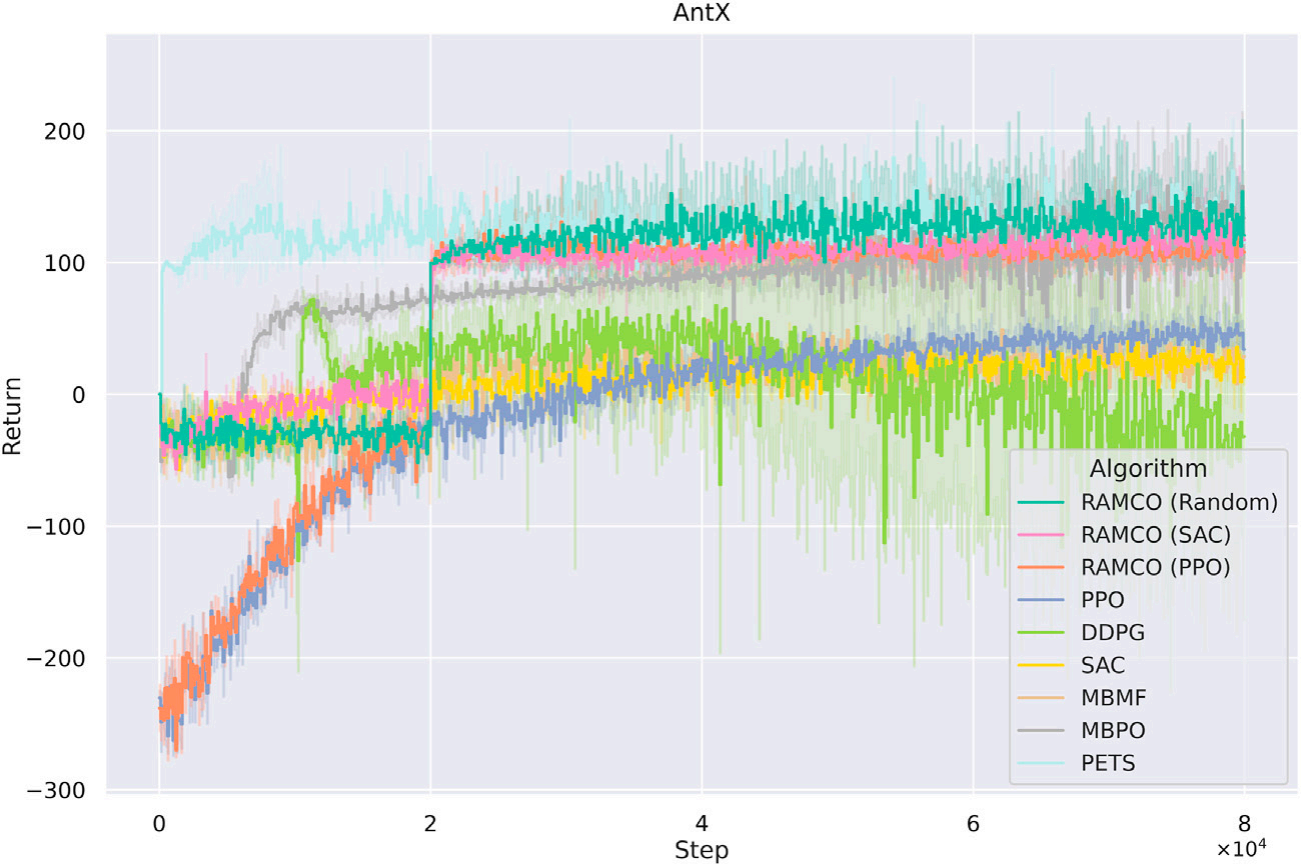
Accumulated rewards from each episode of AntX for different algorithms. RAMCO (SAC) and RAMCO (PPO) stand for using SAC and PPO as training data generators in our RAMCO control framework, respectively. These two model-free agents are trained from scratch. RAMCO (Random) means RAMCO with random data for training of the dynamics model. Although our method uses $2\times 10^{4}$ steps to warm-up, it can outperform all other methods (except PETS) in its very first controlled iteration. The use of SAC as the warm-up policy can further enhance the quality of results when compared to PPO warm-ups. Our methods also show their advantage of low variance.

We test our RAMCO method with different warm-up policies (SAC, PPO and random) on our AntX model with the hyperparameters shown in the supplementary material, and these results are shown in Figure 8. All the result data are an average of three runs. We compare our results with RL algorithms listed below:

- **Proximal Policy Optimization (PPO) (** Schulman et al., 2017): PPO is one of the most popular and successful model-free RL algorithms. As introduced in Section 2, essentially it is a policy-gradient-based RL method.
- **Deep Deterministic Policy Gradient (DDPG) (** Lillicrap et al., 2015): DDPG is another model-free RL algorithm with an actor-critic structure.
- **Soft Actor-Critic (SAC) (** Haarnoja et al., 2018): SAC adds entropy as part of its objective function for better exploration. It reports better data efficiency than DDPG on MuJoCo benchmarks.
- **Model-Based Model-Free hybrid (MBMF) (** Nagabandi et al., 2018): The model-based part of the method MBMF is implemented. It is an deterministic model-based method using random shooting method as the optimiser.
- **Model-Based Policy Optimization (MBPO) (** Nagabandi et al., 2018): MBPO uses a probabilistic dynamics model to generate additional data to a replay buffer for training a SAC-based model-free agent. It is reported that this can highly improve data efficiency.
- **Probabilistic Ensembles with Trajectory Sampling (PETS) (** Chua et al., 2018): PETS is a recent pure model-based method. It uses deep neural networks with ensembles to model the environment dynamics taking the uncertainty in consideration, and does open-loop planning based on this model.

In addition, the first four methods use the implementation from TF2RL package (Kei Ohta, 2020) with all default hyperparameters. MBPO uses its official implementation and PETS uses the implementation from (Vuong, 2020). We run all these methods for $8\times 10^{4}$ time steps. It can be obtained from the figure that although our method uses $2\times 10^{4}$ steps to warm-up (generates training data for the dynamics model without risk-awareness), they can produce very competitive results in this case in terms of both average and variance. RAMCO based on random warm-up data and SAC warm-up policy outperform all other methods (except PETS) in its very first risk-aware controlled iteration. The SAC-based RAMCO shows a lower variance compared to PETS, while the random-based RAMCO shows a competitive average. The PPO-based RAMCO method has a very stable performance after the warm-up phase, which could be a very useful property in risk-sensitive applications. It also shows a higher return compared to all the model-free methods (PPO, DDPG, and SAC) and the model-based method MBMF. In addition, the returns from DDPG algorithm are unstable, with sudden learning drops which could lead to fatal behaviors in real-world applications, but the algorithm surpasses our solution after $4.5\times 10^{5}$ (as shown in the full-sized figure at the supplementary material).

**FIGURE 9:**
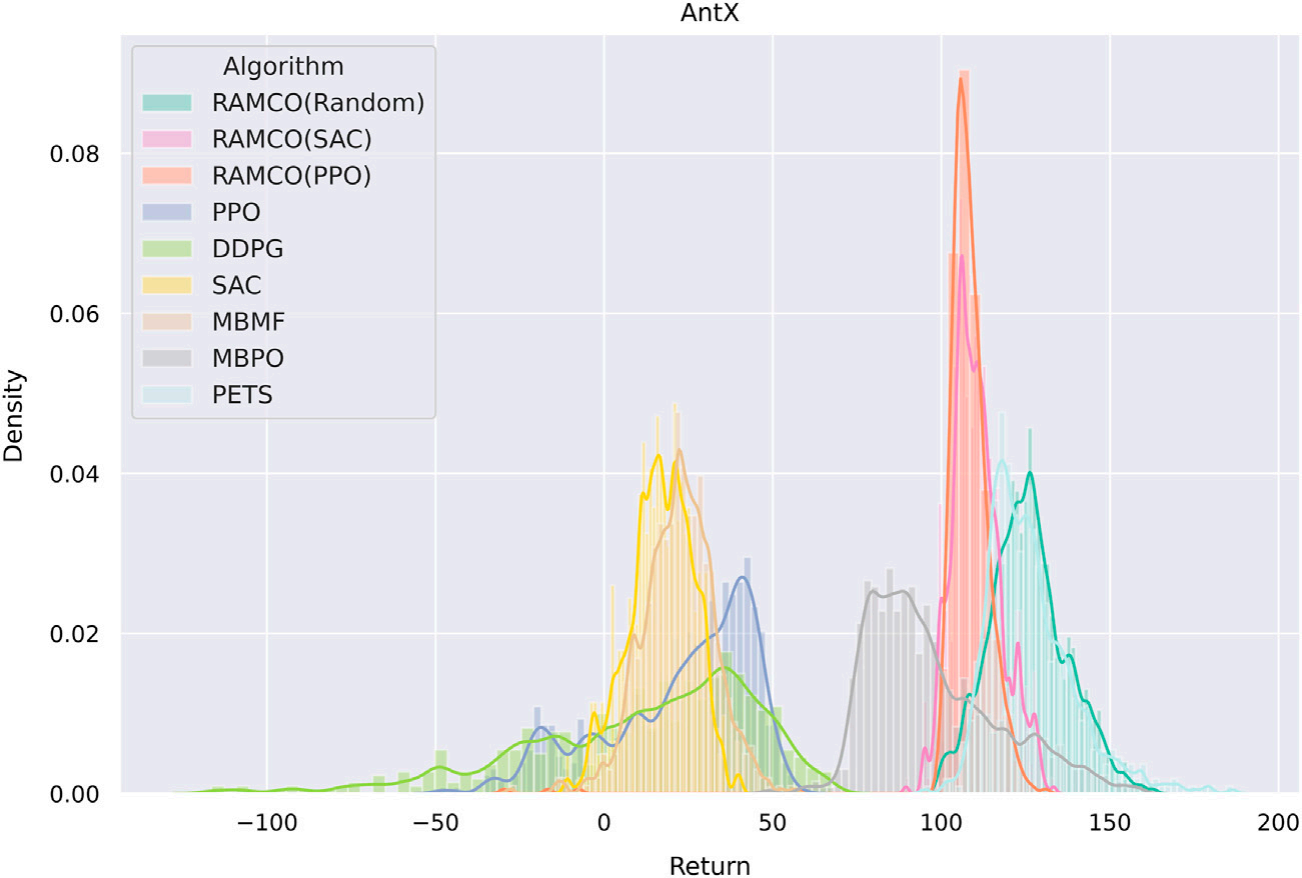
A histogram showing the distribution of final returns among different algorithms. Data of the warm-up phase of RAMCO and the corresponding period of other methods are dropped. We can see from the figure that not only the average of our returns is outstanding, but also the variance. While the random-based RAMCO has shown an exceptional average return, the PPO-based RAMCO shows the lowest variance among all the algorithms, which could be a very useful property in risk-sensitive applications. The SAC-based RAMCO has a variance between the two.

It is noticed that some specially designed risk-sensitive metric is not used in the comparison but the final returns are shown since this is fairer for other algorithms. Furthermore, we consider that users will focus more on the final performance in their applications and this also meets with our assumption in Section 3 that the negative of total discounted future reward is equivalent to the value of loss. In order to highlight the superior of our risk-aware framework, we plot a histogram showing the distribution of final returns (after the warm-up phase of RAMCO and the corresponding period of other methods) among different algorithms in Figure 9. It can be obtained from the figure that not only the average of our returns is outstanding, but also the variance. While the random-based RAMCO has shown an exceptional average return, the PPO-based RAMCO shows the lowest variance among all the methods. RAMCO based on SAC warm-up policy produces a variance between the two.

Compared to another state-of-the-art model-based method PETS, it is observed from Figures 8, 9 that our methods are highly competitive with respects to the average and variance. While the random-based RAMCO shows a similar average return to PETS, the use of SAC and PPO as the warm-up policy can further reduce the variance and hence risk. This means that the choice of the back-end warm-up policy can be used for trading between risk and return. This feature comes from our risk-awareness nature and more flexible framework compared to PETS. On the one hand, our CVaR decision-making mechanism tends to perform conservative strategies; on the other hand, we can choose the back-end warm-up policy (random policy, SAC, or PPO) to meet users’ requirement of performance and robustness. We will discuss more on the differences between our method and PETS in Section 7.1 and our advantages on generalization and risk aversion over PETS in sections 7.2 and 7.3.

### 6.3 RAMCO on Eidos

**FIGURE 10:**
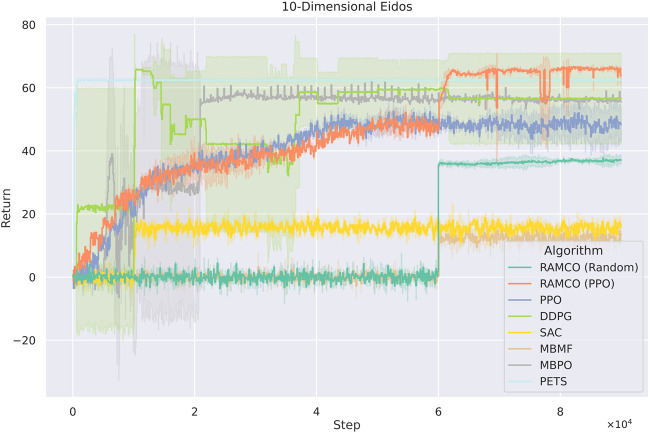
Training curves for Eidos with 10 state dimensionality. The PPO-version of RAMCO outperforms all other methods after $6\times 10^{4}$ steps of warm-up. Among model-free algorithms, DDPG produces the closest result to ours but has a very unrobust training behavior and worse final return. Our RAMCO method (PPO-warm-up version) surpasses all other model-based methods including PETS and especially show overwhelming superiority to MBMF.

Based on the fact that Eidos is a very efficient method to evaluate RL algorithms, which is shown in the supplementary material, we benchmark different algorithms on Eidos with state dimensions of 10, $10^{2}$, and $10^{3}$. All the algorithms run for $8\times 10^{4}$ steps, and all the result data are an average of three trials. In the 10 dimensions case (Figure 10), the returns of RAMCO based on PPO warm-up policy surpasses that of all other methods after $6\times 10^{4}$ steps of warm-up. Among model-free algorithms, DDPG produces the closest result to ours but has a very unrobust training behavior and worse final return. Our RAMCO method (PPO-warm-up version) also surpasses all other model-based methods including PETS and especially show overwhelming superiority to MBMF.

**FIGURE 11:**
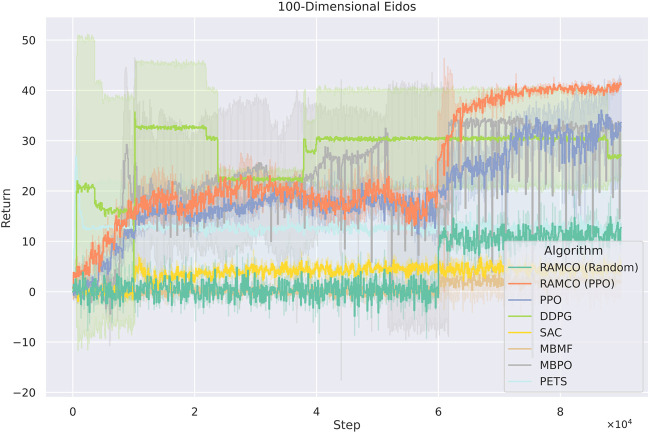
Training curves for Eidos with 100 state dimensionality. The PPO-version of RAMCO continuous to show its advantages over all other model-based and model-free methods. In this case, model-free methods begin to show its advantages on scalability while other model-based methods start to show their shortage. However, RAMCO based on PPO-generated training data still outperforms other state-of-the-art model-free methods including PPO, DDPG and SAC and model-based methods including PETS, MBMF and MBPO after the warm-up phase.

In the 100 dimensions case (Figure 11), PPO-version of RAMCO still shows advantages over all other model-based and model-free methods. In this case, model-free methods such as PPO begin to show its advantages on scalability while other model-based methods start to show their limit of applications. However, RAMCO based on PPO-generated training data still shows a jump in its training curve after the warm-up phase, which makes it outperforms other state-of-the-art model-free methods including PPO, DDPG and SAC and model-based methods including PETS, MBMF and MBPO. The random-based RAMCO seems to have a lower return than the one based on PPO, while still produces a competitive result compared to SAC, PETS, and MBMF.

**FIGURE 12:**
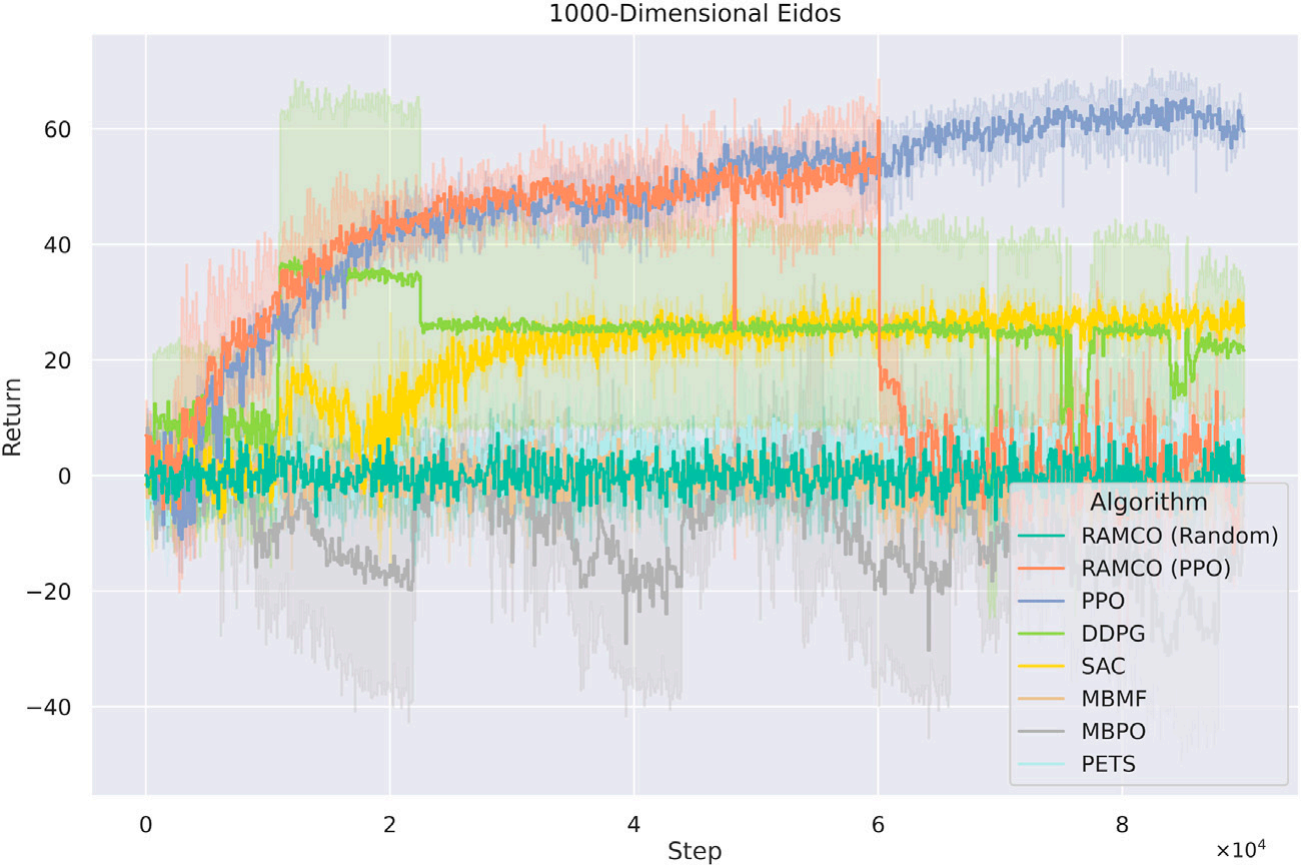
Training curves for Eidos with 1,000 state dimensionality. RAMCO (Random) shows performance similar to PETS, which are still better than the model-based method MBMF and hybrid method MBPO. The PPO-warm-up version of RAMCO reached its highest returns during the warm-up phase, and its performance degrades afterwards. Therefore, PPO-warm-up RAMCO excels while dealing with scalability with its flexible control framework in real-world applications where the back-end warm-up policy can be adopted directly.

As for the cases with 1,000 dimensions (Figure 12), RAMCO (Random) shows performance similar to PETS, which are still better than the model-based method MBMF and MBPO. The PPO-warm-up version of RAMCO reached its highest returns during the warm-up phase, and its performance degrades afterwards. While RAMCO excels in low dimensionality data efficiency, even when the warm-up is taken into consideration, it still suffers from the curse of dimensionality. Nonetheless, its flexible control framework makes it a good candidate for real-world applications by directly deploying the underneath warm-up policy for solving problems.

Overall, in the 10-dimension case, our RAMCO method (with a back-end PPO policy) shows its advantages on both performance and robustness over all other model-based and model-free methods. In the case with $10^{2}$ dimensionalities, RAMCO based on PPO warm-up data still produces competitive results, especially compared with all other model-based methods, which are all suffering through scalability. While the state dimensionality is increased to $10^{3}$, the PPO-warm-up RAMCO is still a promising tool in a real-world application, where the back-end warm-up policy can be adopted. Based on these results, we will further discuss the value of our methods in respect of data efficiency, generalization (robustness and scalability), and risk aversion in the next section.

## 7 Discussion

This paper proposes a novel algorithm called RAMCO. It uses a probabilistic dynamics model to model the transition function whose initial training data can be produced by a random policy or model-free agents. Within this method, we use CVaR as a metric to evaluate the predicted trajectories, and once the optimum action sequence is found by CMA-ES, the first action of the sequence is taken to interact with the environment. To be consistent with our comparisons, we also propose a novel RL environment called Eidos. In short, Eidos is a pseudo environment consisting of two randomly initialized deep neural networks: one representing the ground-truth dynamics model and one regarded as a reward function. After showing the effectiveness of this Eidos environment, we show that our method achieves a remarkable trade-off between data efficiency, generalization, and risk aversion.

### 7.1 Data Efficiency

Data inefficiency can lead to a heavy human workload during real-world applications, which ironically contradicts the original motivation behind using learning algorithms. However, such inefficiency can still be found in most model-free algorithms (Chatzilygeroudis, 2018). As we show in Figures 8,10, all the model-free algorithms, including PPO, DDPG, and SAC, do not perform well in these two cases, mostly because of their data-inefficiency: $8\times 10^{4}$ iterations are not enough for a model-free algorithm to find an convergent solution in such dimensionalities. MBPO can be regarded as an SAC algorithm with a dynamics model added to it, and hence an evident advantage over SAC and other model-free methods.

The model-based method PETS benefits from its probabilistic dynamics model and shows a competitive result when compared to our RAMCO method. This success presented by PETS can be explained from its nonconsideration of risk. First, PETS does not distinguish the warm-up phase and application phase and has a higher update frequency of the dynamics model, which makes the improvement of policies more aggressive. However, this also leads to a higher risk of being constrained to a local optimum, which can be proven by the results of the 10-dimensional case, where the returns of our methods surpass the ones from PETS. The second reason for the advantage of PETS in the AntX case is that its evaluation only takes into consideration the average returns. Compared to the conservative policy of RAMCO, PETS performs a risky strategy which outputs higher mean returns but also a higher variance. We will further discuss the negative impact of this trade-off in Section 7.3. As for another model-based method MBMF, the random-shooting optimiser used for the MPC is not enough to find an optimum solution effectively in all cases. Overall, our method shows its advantage in data efficiency as an MBRL algorithm, which makes it competitive on the AntX and 10-dimensional Eidos environments.

### 7.2 Generalization

The generalization of an RL algorithm can be characterized in two aspects: robustness and scalability, both of which attract less attention from the MBRL communities, but still crucial for real-world application. When in situations where humans themselves cannot predict what the best policies/actions are, RL algorithms require a certain degree of insensitivity to hyperparametric choices and environment complexity. In this sense, our proposed Eidos environment is an efficient tool to evaluate the generalization of RL algorithms, and RAMCO still proves to be efficient following this metric.

As it can be seen from Figures 11, 12, model-free RL algorithms have natural advantages on scalability. For instance, DDPG begins to show a higher return than RAMCO on the 100-dimensional Eidos environment and all the model-free methods achieve higher returns than RAMCO based on random rollouts on the 1000-dimensional Eidos. Conversely, scalability is a common problem for model-based methods focusing on data efficiency. Since it is challenging to learn a precise dynamics model in a complex environment even with neural networks, the model bias can often lead to failed decision making. This can be proven by the last two cases, where the problem dimensionality expands to 100 and 1,000. As we can see, while the dimensionality of the environment increases, model-based methods (including PETS and MBMF, and hybrid methods like MBPO) begin to suffer from model bias and hence produce much worse results than model-free methods. Other model-based methods, such as black-box optimisers (Hansen et al., 2015) to find a closed-loop policy (Chatzilygeroudis et al., 2017) or using policy gradient to find an optimum policy with respect to a Bayesian neural network (Gal et al., 2016), suffer from time complexity, so they mostly focus on simple tasks like cart-pole-swing-up or double-pendulum swing-up with lower dimensionalities (typically less than 10). As mentioned in Section 2, modeling the transition function with Gaussian Process (Deisenroth and Rasmussen, 2011; Kamthe and Deisenroth, 2018) is also a barrier for applying some MBRL methods to more complex environments.

As for RAMCO, we solve this problem with the flexibility of the warm-up policy. In the case of 100-dimensional Eidos, the RAMCO method based on PPO rollouts can still produce successful results compared with other methods, supported by our MC-dropout-based probabilistic dynamics model. In the case of 1000-dimensional Eidos, although our methods also suffer from model bias, in practice, we can directly adopt the warm-up policy to make up for this generalization problem of model-based methods. Therefore, the PPO-rollouts version is recommended for all environments with any dimensionality.

### 7.3 Risk Aversion

When compared to other methods, RAMCO is the only one taking calculated risks on its trials and hence producing much smoother training curves in most cases. By preventing drastic fluctuations, such as shown by the training returns produced by DDPG in the AntX case, it shows its excellence towards real-world application to avoid catastrophic loss. Compared to PETS on the AntX environment, although the PPO-version of our strategies causes a lower mean return, it shows a lower variance in the controlled phase, which is also meaningful for risk-sensitive real-world applications where expectable output is required.

In term of risk-sensitive control, most of the prior works are based on assuming a stochastic MDP (Tamar et al., 2014; Roy et al., 2017; Derman et al., 2019) and trying to reduce risk due to aleatory uncertainty, as we have introduced in Section 2. Since we believe that the risk assessment should take into consideration the epistemic uncertainty within an engineering application, we focus on the epistemic risk and maintain a regular environment setting. Depeweg et al. (Depeweg et al., 2017) decompose aleatory and epistemic uncertainty and propose a risk-sensitive RL algorithm to attain a balance between expected reward and risk. However, their consideration of risk is solely with respect to individual rewards, instead of the total return of each episode. Work with a very similar risk-control objective to ours can be found in (Eriksson and Dimitrakakis, 2020). There, the goal is to control the epistemic risk by a model-free RL algorithm and utility function. However, they only show experimental results on Gridworld and option pricing but not locomotion control. In addition, since it is based on a model-free structure, the data inefficiency limits its real-world application, which is inconsistent with the motivation of using a risk-sensitive model. In order to solve the problem of data inefficiency, there are also works on risk-sensitive batch-RL algorithms (Thomas et al., 2015; Levine et al., 2020), which return incremental policies based on historical data with probabilistic guarantees about the quality of generated policies. Their problem setting is closer to applications such as marketing and therapies.

It can be seen that there exists a broad diversity within the field of risk-sensitive RL. Therefore, specific settings and improvements of our Eidos environment should be our further work when building upon a general benchmark for risk-aware RL. At the same time, instead of using a constant α parameter for the CVaR component at RAMCO, an alternative path for our future work is to study the effects of this variable on the risk-sensitive performance on different Eidos settings. Furthermore, based on the flexibility of the Eidos environment, interesting and valuable experiments involving concepts of epistemic value, epistemic action, and active perception can be further designed in future work. The epistemic cost in these experiments can be further evaluated on both warm-up and application phases, which could be a significant metric in real-world risk-sensitive applications.

## 8 Conclusion

In this work, we propose an MBRL algorithm called RAMCO and an RL environment called Eidos, both motivated by the need from real-world applications to quickly converge to an optimal solution without incurring in catastrophic failures. While RAMCO is a risk-sensitive and data-efficient algorithm, Eidos is an environment which allows us to simulate a multitude of unknown/multi-dimensional problems which could be faced by an RL agent in the real-world. Compared to other works in MBRL methods (Deisenroth and Rasmussen, 2011; Nagabandi et al., 2018; Chua et al., 2018), we assign more attention to risk-awareness, robustness, and scalability; while compared to other works in robust MDP (Tamar et al., 2014; Roy et al., 2017; Derman et al., 2019), we innovate by considering epistemic uncertainty and data efficiency. We empirically show that the dynamics model in our RAMCO method can meet the requirements of our setting and the overall performance is competitive to state-of-the-art model-based and model-free RL algorithms. The results are encouraging as a step towards bringing RL into safe industrial applications.

## 9 BROADER IMPACT

Robots in our daily life are gradually transitioning from a utopic “sci‐fi” concept to a concrete reality, and the capacity to adapt within very few trials to disturbances and malfunctions is crucial. Our work concentrates on risk-awareness and data efficiency, specifically, to address this problem. Unlike other works which rely on the abundance of data to find solutions (big data approaches), our solution deviates from this trend to follow a micro data approach. The reason for this is because trials can be very expensive, and failures can result in property damage (robotics) or loss of lives (therapeutics).

Our proposed algorithm excels on problems with less than 100 dimensions, finding excellent solutions right in its first trial after finishing its warming up phase. Although it has a higher computational cost per episode than its counterparts (which is convenient, in juxtaposition to the very nature of problems that it aims to solve, as “expensive trials” shouldn’t be quickly repeated), the exponential advances in computer architectures and cloud computing will make such strategies more preferable in the long run, quickly taking in consideration real-world inputs to output better strategies to physical machines directly.

## Data Availability

The original contributions presented in the study are included in the article/Supplementary Material, further inquiries can be directed to the corresponding author.
