# Supplementary material for: Risk-Aware Model-Based Control
Source: Front Robot AI. 2021 Mar 11;8:617839. doi: 10.3389/frobt.2021.617839 (PMC7990789; doi:10.3389/frobt.2021.617839)
Supplement: Supplementary file 1 [file datasheet1.pdf]

Supplementary Material

1 ADDITIONAL EXPERIMENTAL DETAILS

1.1 Benchmark environments

We briefly summarise all the benchmark environments used in the experiments and the AntX model in the Tab. S1.

1.2 Hyperparameters and implementation

Some general hyperparameters are set according to Tab. S2 among all environments. Beside, the size of initial training set (warming-up data) is 2×10^4 steps for controlling AntX, and 6×10^4 steps for solving Eidos. To produce the multi-environment validation table, size of training set is 600 rollouts and which of validation set is 200 rollouts. In addition, all our code including the RAMCO algorithm and experimental environments are open-source on `https://github.com/Chenaah/RAMCO`.

1.3 Computational cost analysis

For the flexibility of our RAMCO framework, the computational cost is highly dependent on hyperparameters and implementation. In the pseudocode Alg. 1 of our method, evaluation of actions sequences in each iteration m in loop M is parallelised by GPU computing, and loop N is parallelised by multi-processing. Based on these code optimisation, the number of generations of CMA-ES G is one of the most critical parameters for time complexity control. When we set G=5 in our experimental section, it takes around 71 hours to complete an 8×10^4-step AntX experiment on a computer with an i7-8700 CPU and a GeForce GTX 1060 graphic card. With the same number of generations and setup of hardware, it takes 100 hours to finish a 9×10^4-step 10-dimensionality Eidos experiment, 126 hours to finish a 9×10^4-step 100-dimensionality Eidos experiment, and 359 hours to finish a 9×10^4-step 1000-dimensionality Eidos experiment. It is noticed that these time-costs highly depend on the hardware conditions. In addition, to decrease the computational cost in real-world applications, users can decrease the iteration number G to sufficient for the specific task.

2 ADDITIONAL EXPERIMENTAL RESULTS

2.1 Dynamics model

Here we visualise the predicted results of the trained dynamics model on more environments in Fig. S1. The trained dynamics model used in this visualisation is based on random warming-up data.

2.2 RAMCO on AntX

Base on the result figure Fig. 8 in the main paper, We run the model-free methods for more steps and show the results in Fig. S2.

2.3 Eidos analysis with state-of-the-art RL algorithms

Before testing our RAMCO algorithm on the Eidos environment, we first have a case study on a state-of-the-art model-free algorithm PPO about its behaviour on our Eidos environment. We fix the action dimensions of the environment as 10 and vary the state dimensions as 10, 10^2, 10^3, 10^4, and 10^5. We assume the environment is fully-observable and additional Gaussian noise is added to the observation with a signal-to-noise ratio (SNR) of 60. With the only existing terminal condition, the maximum number of steps for each episode is 100. These settings are the same as of the experiments in section 6.3. The results of this case study are shown in Fig. S3. It can be observed that the learning curve becomes nosier, and it is also harder to obtain convergent results when the environment becomes more complex. When the

Table S1. Environments used in the experiment.

Environment	Simulator	Description	Image	State-Space	Action-Space
AntX	MuJoCo	A 3D four-legged robot modified from Ant-v2.	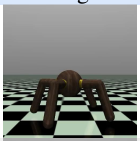	29	8
Ant-v2	MuJoCo	A 3D four-legged walking robot, initially appeared in Schulman et al. (2016).	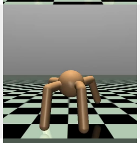	111	8
Inverted-Double-Pendulum-v2	MuJoCo	Balance a pole on a pole on a cart.	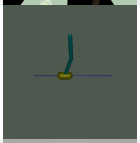	11	1
HalfCheetah-v2	MuJoCo	Make a 2D cheetah robot run.	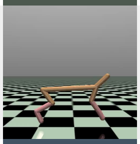	17	6
Hopper-v2	MuJoCo	Make a 2D robot hop.	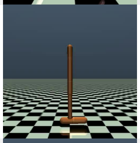	11	3
Walker2d-v2	MuJoCo	Make a 2D robot walk.	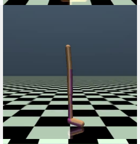	17	6
Swimmer-v2	MuJoCo	Make a 2D robot swim.	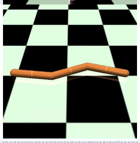	8	2
AntBulletEnv-v0	PyBullet	Similar to Ant in MuJoCo but is more massive, encouraging more legs on the ground.	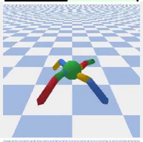	28	8
Half-Cheetah-BulletEnv-v0	PyBullet	Similar to the HalfCheetah in MuJoCo.	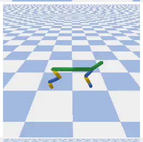	26	6
Minitaur-BulletEnv-v0	PyBullet	A quadruped robot on flat ground, initially used in Tan et al. (2018)	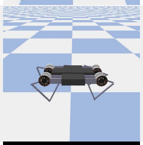	28	8
Lunar-Lander-Continuous-v2	Box2D	Navigate a lander to its landing pad.	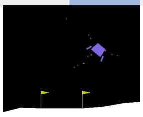	8	2
Bipedal-Walker-v3	Box2D	Train a bipedal robot to walk.	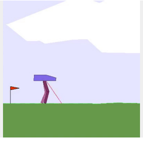	24	4

Table S2. Some default hyperparameters

Hyperparameter	Value
Size of training set (number of random rollouts)	600
Size of validation set (number of random rollouts)	200
Batch size	512
Dropout probability p	0.9
Size of hidden layer	500
Learning rate	0.001
regularization parameter λ	0.01
Maximum generations G	5
Transitions sample K	1
Trajectories sample M	10
Planning horizon T	5

dimension of the state is only set as 10, the agent can reach a return of around 50 at the very beginning, and when the dimension of state is tuned up to be 10^5, it only obtains a return of less than 14 after 10^6 steps of training.

To further highlight the impact of different environment parameters on the algorithm's performance, we vary the state dimension from 100 to 1000 with a step size of 100 while varying the action dimension from 10 to 100 with a step size of 10. We run the PPO algorithm on the Eidos environment for 10^6 steps for each of these parameters setting. We calculate the mean and variance of returns during the training process with different settings and show it as a heatmap as Fig. S4. As shown in the figure, we can not find a strong pattern beyond the mean returns, since the range of parameters is relatively narrow, and there exists a trade-off between agents' model mismatch and environment complexity. However, from the Fig. S4.B we can infer that complex environments (simulated complexity at our Eidos environment) translate as a higher degree of instability. With this capacity to push learning algorithms to its limit, we claim that Eidos is a very efficient method to evaluate RL algorithms.

REFERENCES

Schulman, J., Moritz, P., Levine, S., Jordan, M. I., and Abbeel, P. (2016). High-dimensional continuous control using generalized advantage estimation. *CoRR* abs/1506.02438

Tan, J., Zhang, T., Coumans, E., Iscen, A., Bai, Y., Hafner, D., et al. (2018). Sim-to-real: Learning agile locomotion for quadruped robots. *Robotics: Science and Systems XIV* doi:10.15607/rss.2018.xiv.010

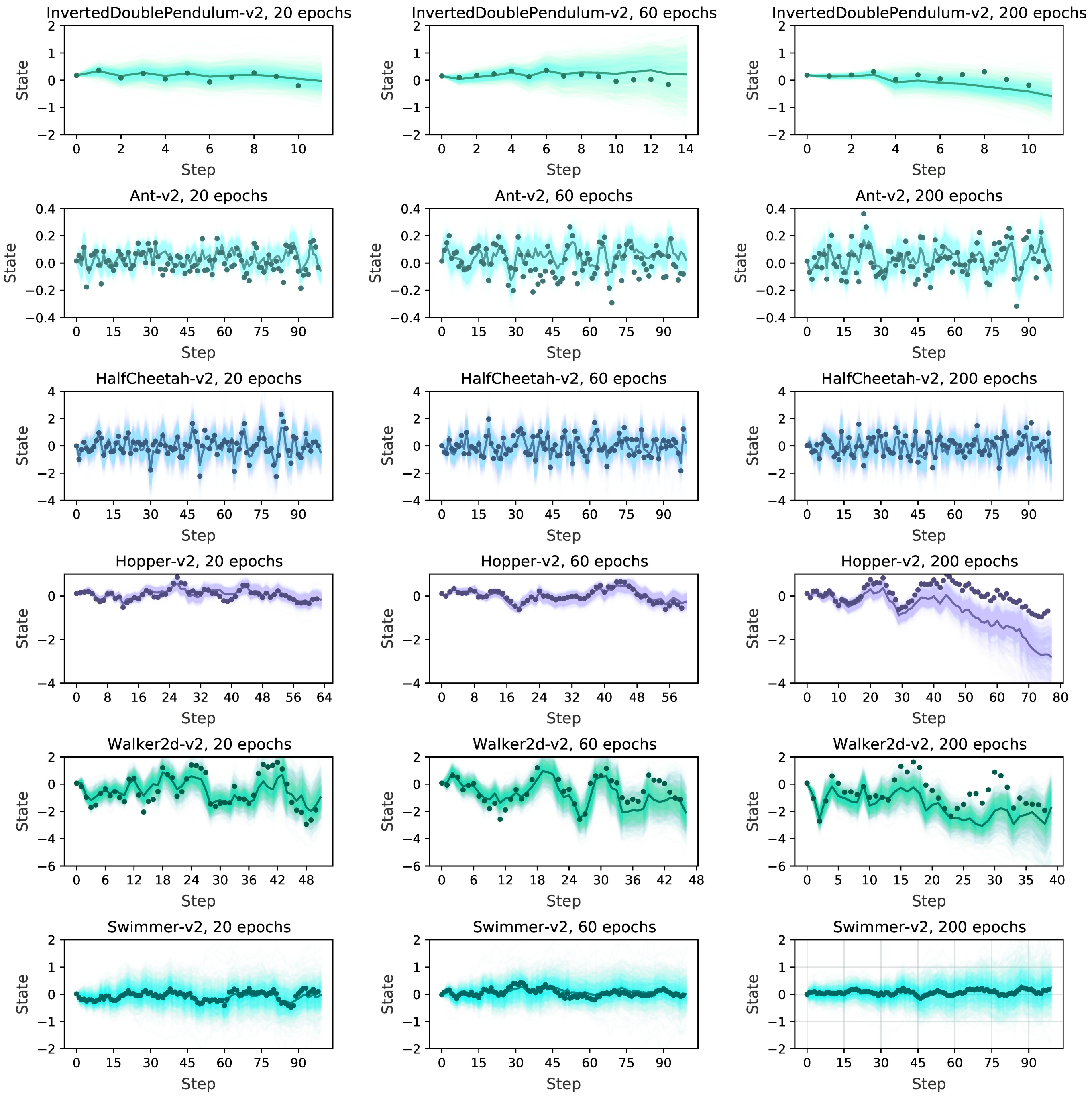

Figure S1. More figures on the predicted trajectories with RAMCO. After a random warm-up we predict states recursively with the dynamics model. Each prediction and predictions average are in light and dark colors, and the baseline in dots.

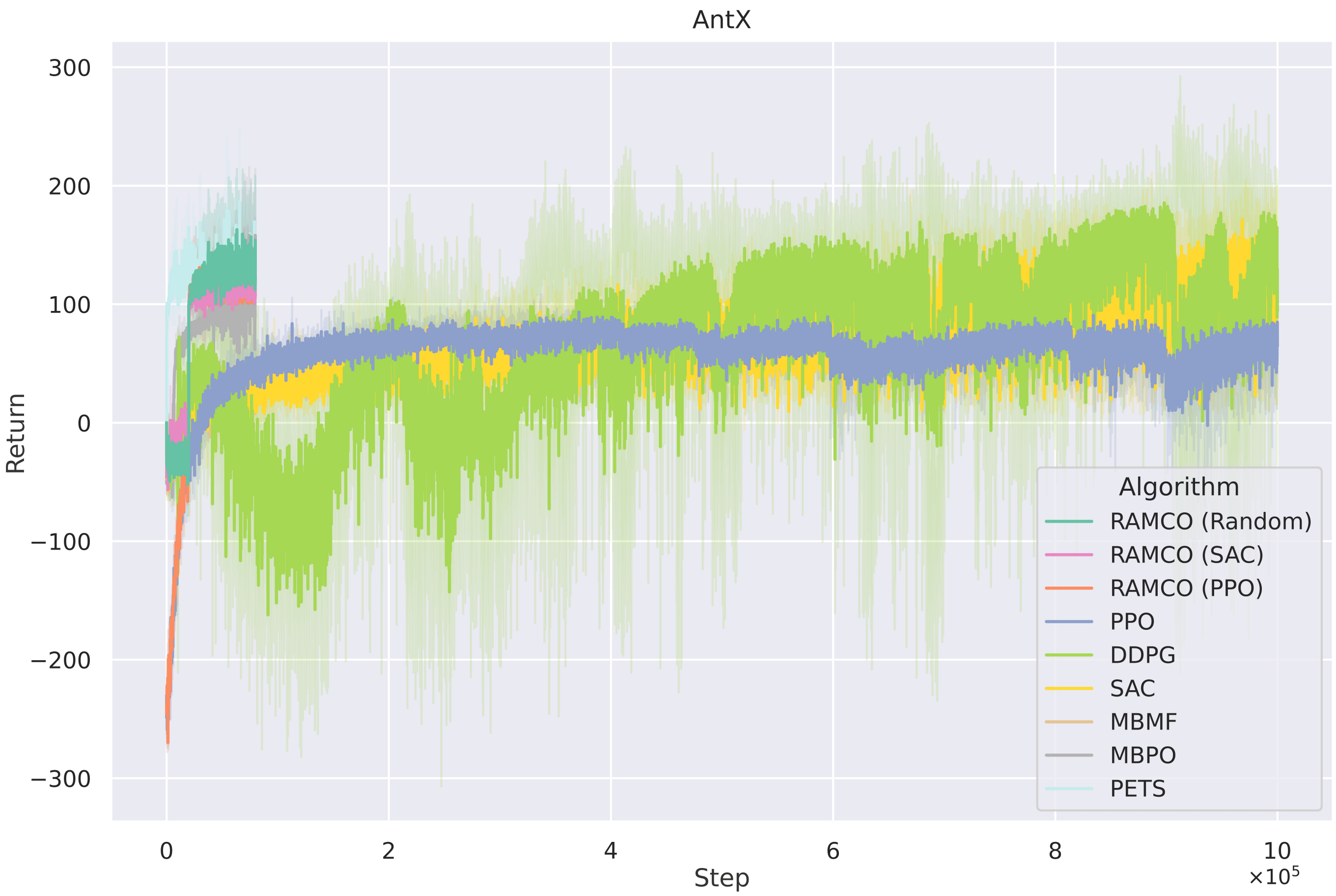

Figure S2. Accumulated rewards each episode of AntX for different algorithms while training other methods for more steps. Compared to our methods (RAMCO with random warming-up data and RAMCO with PPO warming-up policy), DDPG takes more than three times of steps to get our highest score, and SAC takes even more than six times of steps. Although these two model-free methods can find a better policy in term of the highest return, our methods still show their advantages in data efficiency.

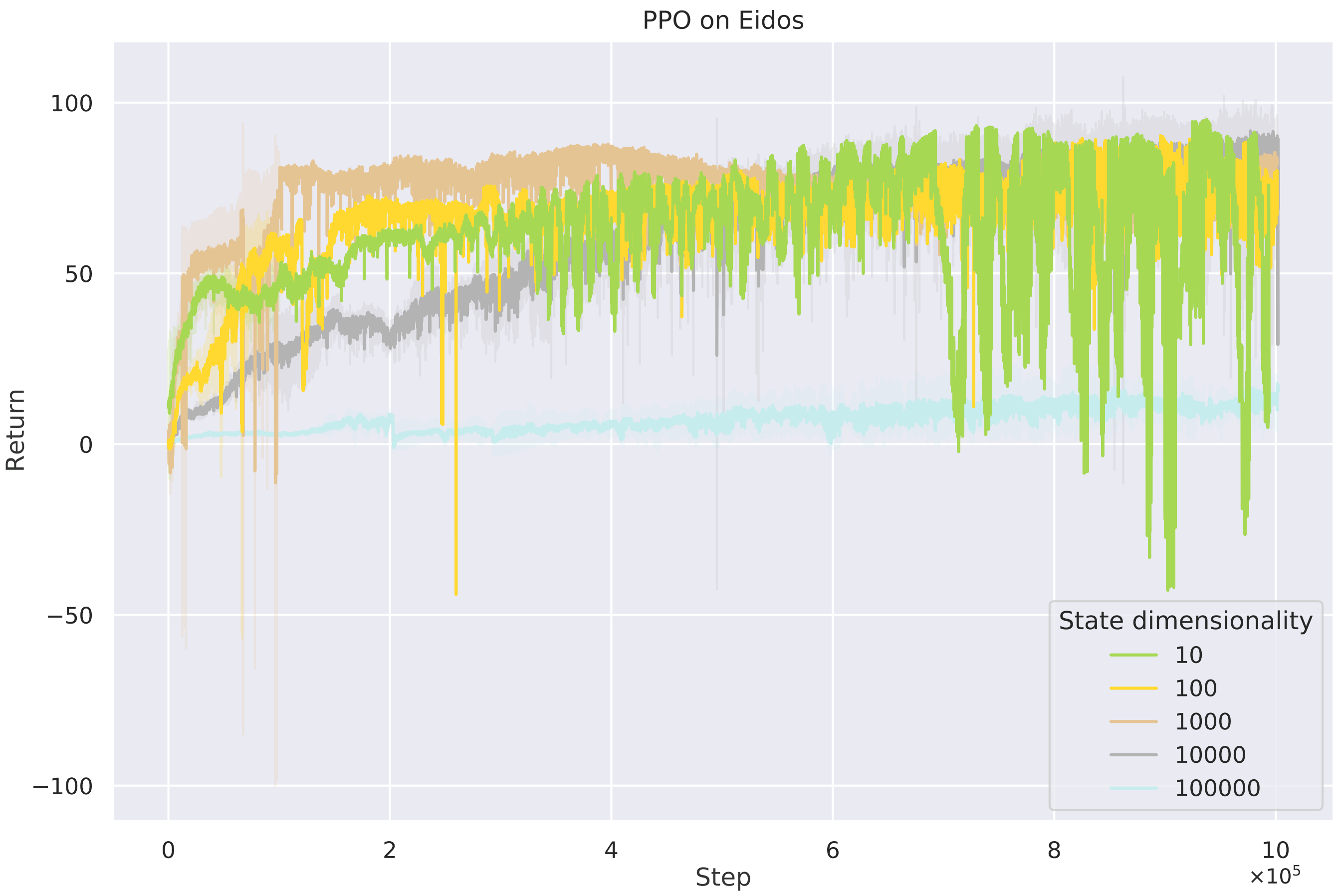

Figure S3. Returns of using PPO algorithm to solve Eidos environments with different state dimensionality. The learning curve becomes nosier, and it is harder to get the convergent results when the environment becomes more complex.

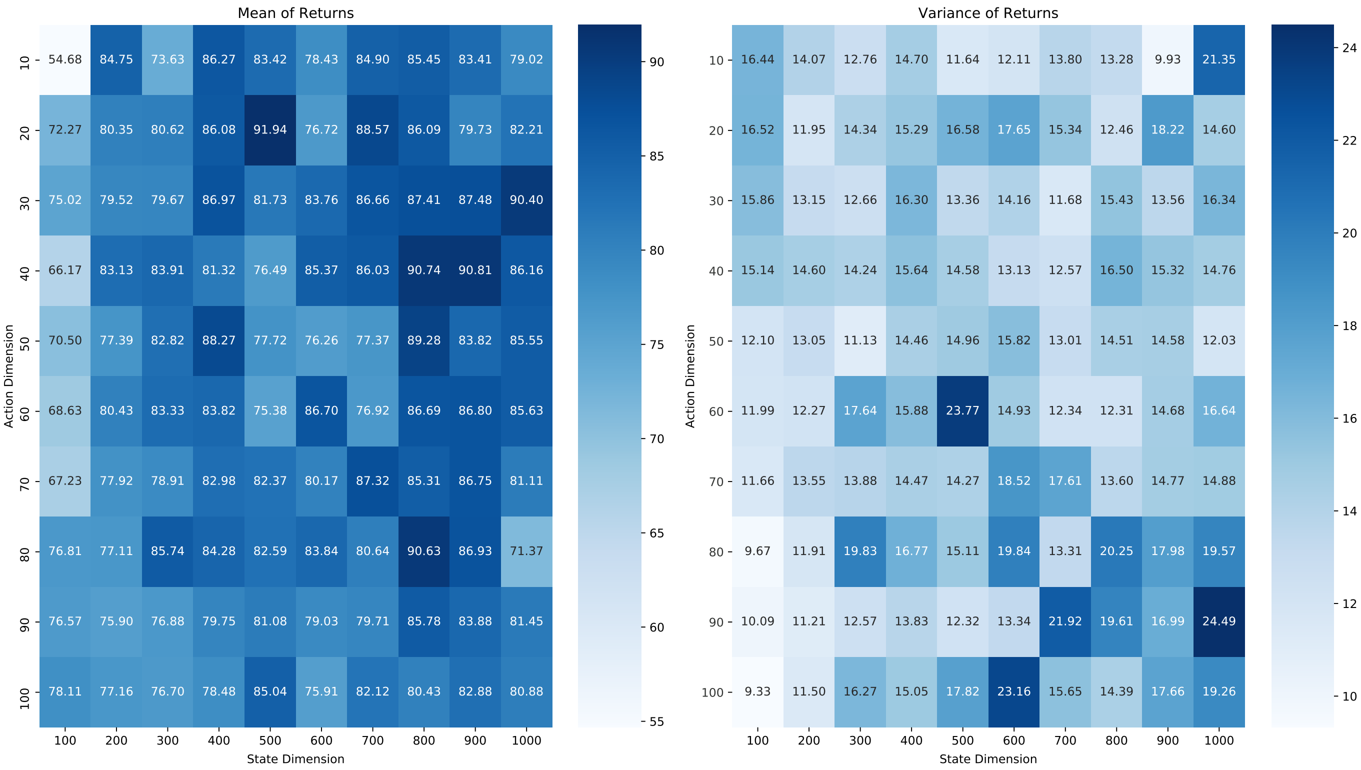

Figure S4. Impact of different environment parameters on the algorithm's performance: **(A)** mean and **(B)** variance of training returns. The variance of training returns shows the effectiveness of using Eidos as an evaluation tool even within a limited parameters range.
